# The burden of tuberculosis and attributable risk factors in Brazil, 1990–2017: results from the Global Burden of Disease Study 2017

**DOI:** 10.1186/s12963-020-00203-6

**Published:** 2020-09-30

**Authors:** Francisco Rogerlândio Martins-Melo, Juliana Maria Trindade Bezerra, David Soeiro Barbosa, Mariângela Carneiro, Kleydson Bonfim Andrade, Antonio Luiz Pinho Ribeiro, Mohsen Naghavi, Guilherme Loureiro Werneck

**Affiliations:** 1 Federal Institute of Education, Science and Technology of Ceará, Rua Francisco da Rocha Martins, S/N, Pabussu, Caucaia, CE 61609-090 Brazil; 2 Epidemiology of Infectious and Parasitic Diseases Laboratory, Department of Parasitology, Institute of Biological Sciences, Universidade Federal de Minas Gerais, Avenida Presidente Antônio Carlos, 6627, Pampulha, Belo Horizonte, MG 31270-901 Brazil; 3 National Tuberculosis Programme, Department of Chronic Infectious Diseases and STI, Secretariat of Health Surveillance, Brazilian Ministry of Health, SRTVN, Quadra 701, Via W5 Norte, Lote D, Edifício PO700, 6° andar, Brasília, DF Brazil; 4 Hospital das Clínicas, Faculty of Medicine, Federal University of Minas Gerais, Avenida Prof. Alfredo Balena, 110, Santa Efigênia, Belo Horizonte, MG 30130-100 Brazil; 5 Institute for Health Metrics and Evaluation, University of Washington, 2301 Fifth Avenue, Suite 600, Seattle, WA 98121 USA; 6 Institute of Studies in Public Health, Federal University of Rio de Janeiro, Avenida Horácio Macedo, S/N, Ilha do Fundão – Cidade Universitária, Rio de Janeiro, RJ 21941-598 Brazil; 7 Department of Epidemiology, Social Medicine Institute, State University of Rio de Janeiro, Rua São Francisco Xavier 524, Maracanã, Rio de Janeiro, RJ 20550-013 Brazil

**Keywords:** Tuberculosis, Burden of disease, Disability-adjusted life year, Brazil

## Abstract

**Background:**

Tuberculosis (TB) continues to be an important cause of fatal and non-fatal burden in Brazil. In this study, we present estimates for TB burden in Brazil from 1990 to 2017 using data from the Global Burden of Diseases, Injuries, and Risk Factors Study 2017 (GBD 2017).

**Methods:**

This descriptive study used GBD 2017 findings to report years of life lost (YLLs), years lived with disability (YLDs), and disability-adjusted life years (DALYs) of TB in Brazil by sex, age group, HIV status, and Brazilian states, from 1990 to 2017. We also present the TB burden attributable to independent risk factors such as smoking, alcohol use, and diabetes. Results are reported in absolute number and age-standardized rates (per 100,000 inhabitants) with 95% uncertainty intervals (UIs).

**Results:**

In 2017, the number of DALYs due to TB (HIV-negative and HIV-positive combined) in Brazil was 284,323 (95% UI: 240,269–349,265). Among HIV-negative individuals, the number of DALYs was 196,366 (95% UI: 189,645–202,394), while 87,957 DALYs (95% UI: 50,624–146,870) were estimated among HIV-positive individuals. Between 1990 and 2017, the absolute number and age-standardized rates of DALYs due to TB at the national level decreased by 47.0% and 68.5%, respectively. In 2017, the sex–age-specific TB burden was highest among males and in children under-1 year and the age groups 45–59 years. The Brazilian states with the highest age-standardized DALY rates in 2017 were Rio de Janeiro, Pernambuco, and Amazonas. Age-standardized DALY rates decreased for all 27 Brazilian states between 1990 and 2017. Alcohol use accounted for 47.5% of national DALYs due to TB among HIV-negative individuals in 2017, smoking for 17.9%, and diabetes for 7.7%.

**Conclusions:**

GBD 2017 results show that, despite the remarkable progress in reducing the DALY rates during the period, TB remains as an important and preventable cause of health lost to due premature death and disability in Brazil. The findings reinforce the importance of strengthening TB control strategies in Brazil through integrated and multisectoral actions that enable the access to prevention, early diagnosis, and timely treatment, with emphasis on high-risk groups and populations most vulnerable to the disease in the country.

## Background

Tuberculosis (TB) is an infectious disease that usually affects the lungs (pulmonary TB), but can also affect other parts of the body (extrapulmonary TB) [1]. Presently, about one-quarter of the world’s population is infected with *Mycobacterium tuberculosis*, but a relatively small proportion (5–15%) will develop TB disease during their lifetime [2].

TB remains a public health problem worldwide, particularly in low- and middle-income countries, despite all efforts to control the disease and recent achievements in reducing incidence and mortality rates [1, 3]. In 2018, there were an estimated 10 million active TB cases [1] and about 45 million disability-adjusted life years (DALYs) worldwide in 2017 [4]. TB is one of the leading causes of death worldwide, with an estimate of 1.5 million TB deaths in 2018 (including 0.25 million people with HIV) [1]. An estimated 1.1 million children (0–14 years of age) fell ill with TB and 205,000 children died from the disease in 2018 [1]. More than 95% of TB deaths occurred in low- and middle-income countries [1].

Like other countries, Brazil has shown a decline in incidence and mortality rates over recent decades, mainly associated with the improvement of population living conditions and the performance of TB control programmes [5, 6]. However, the disease burden continues to be significant in the country [1, 6]. Brazil is one of the 30 high TB burden countries regarding of estimated absolute number of incident TB cases and incident TB cases among people living with HIV [1]. In 2018, about 76,000 new TB cases (including 6700 HIV-positive cases and about 1000 drug-resistant TB cases) and 4500 TB deaths were reported in Brazil, resulting in an incidence rate of 36.6 cases/100,000 inhabitants and a mortality rate of 2.2 deaths/100,000 inhabitants [6]. The geographical distribution of TB is markedly by regional heterogeneities in the country, with incidence TB rates ranging from 22.8 cases/100,000 inhabitants in the Central-West region to 47.7 cases/100,000 inhabitants in the North region in 2018 [5, 6].

Despite the social and health impact of TB in Brazil, few systematic and large-scale studies have been done to assess the fatal and non-fatal burden of TB in the country [7, 8]. The comprehensive assessment and understanding of the trends and levels in TB burden is crucial to track the success of control programmes and to identify remaining intervention challenges seeking to achieve the specific targets of the sustainable development goals (SDGs) until 2030 [3, 9, 10] and World Health Organization (WHO)’s End TB Strategy to end the TB epidemic by 2035 [11]. In this paper, we used results from the Global Burden of Diseases, Injuries, and Risk Factors Study 2017 (GBD 2017) to assess the levels and trends in the burden of TB in Brazil by sex, age group, HIV status, and Brazilian states, from 1990 to 2017. We also report the TB burden attributed to the independent effects of risk factors among HIV-negative individuals, including smoking, alcohol use, and diabetes.

## Methods

### GBD overview

This study has been conducted as part of the GBD study, coordinated by the Institute for Health Metrics and Evaluation (IHME; http://www.healthdata.org) at the University of Washington, USA. The GBD study is a systematic and scientific effort to quantify the comparative magnitude of health loss due to diseases, injuries, and risk factors by sex, age group, and location over time [4]. The GBD study uses as the main population health metric the disability-adjusted life years (DALYs), a summary measure of health loss due to both fatal and non-fatal disease burden [4]. DALYs are estimated by summing up the years lived with any short term or long-term disability (YLDs) and years of life lost (YLLs) due to premature mortality for a given cause [4, 12, 13]. One DALY is equivalent to one healthy year of life lost due to a specific disease or injury [4].

GBD 2017 provides consistent estimates of health lost for 359 diseases and injuries and 84 risk factors for 195 countries and territories, some of which were estimated at the subnational level, including Brazil [4, 14]. For each cycle of the GBD study, the entire time series is re-estimated to incorporate new data and methods. Thus, the GBD 2017 results supersede all previous GBD results [15]. A detailed description of general methodological approaches of GBD 2017 and the specific methodology used to estimate TB burden has been described elsewhere [12–14, 16]. In this study, we used data and estimates from the GBD 2017 study to explore the TB burden by HIV status (HIV-positive individuals, HIV-negative individuals, and HIV-negative and HIV-positive combined) in Brazil from 1990 to 2017.

### Case definition of tuberculosis

The case definition of TB includes all forms, including pulmonary and extrapulmonary TB, which are bacteriologically confirmed or clinically diagnosed [3, 10]. The GBD category of TB is defined and identified according to the International Classification of Diseases (ICD)-9 codes 010–19.9, 137–137.9, 138.0–138.9, 320.4, and 730.4–730.6, and the ICD-10 codes A10–19.9, B90–90.9, K67.3, K93.0, M49.0, and P37.0 [3, 12, 13]. For TB-HIV, the ICD-10 code is B20.0 [3, 10].

### Geographical location and time period

Brazil, officially called the Federative Republic of Brazil, is the South America’s largest country (total territory of 8.5 million km^2^) and has an estimated population of 210.1 million inhabitants in 2019 [17]. The country is divided politically and administratively into 27 federative units (26 states and the Federal District) and 5570 municipalities, grouped into five geographic macro-regions (South, Southeast, Central-West, North, and Northeast). In this study, we present results for TB burden at the national level (entire country) and for all federative units, herein simply named as states.

GBD 2017 estimated cause-specific burden for the years 1990–2017 [4, 12–14]. In this study, we focus on burden estimates for 2017, with reference to changes from 1990. All GBD 2017 results for all years and by location, including Brazil and its 27 states, can be explored further in dynamic data visualizations at http://vizhub.healthdata.org/gbd-compare and http://ghdx.healthdata.org/gbd-results-tool.

### Data sources and processing

The GBD 2017 data sources and analytical process for general mortality and morbidity estimates and for specific analysis of TB burden among individuals who were HIV-positive and HIV-negative have been detailed elsewhere [3, 10, 12, 13]. For Brazil, the main mortality data source used in GBD study was the Brazilian Mortality Information System (*Sistema de Informações sobre Mortalidade – SIM* in Portuguese) database, adjusted by other national and international sources [18–21]. Vital registration data were adjusted and corrected for mortality completeness and redistribution of garbage codes (assignment of causes of death that could not or should not be classified as the underlying cause of death, including ill-defined codes and the use of intermediate causes) to more precise underlying causes of death using GBD algorithms and misclassified HIV deaths (i.e., deaths caused by HIV being assigned to other underlying causes of death, such as TB or diarrhea, because of stigma or misdiagnosis) [3, 10, 13]. GBD 2017 used the Cause of Death Ensemble model (CODEm) strategy to generate estimates of TB deaths among HIV-negative individuals by location, age group, sex, and year [3, 10, 13]. The CODEm approach evaluates a large number of potential models that apply different functional forms (mixed-effects models and spatiotemporal Gaussian process regression models) to mortality rates or cause fractions with varying combinations of predictive covariates and constructs an ensemble model based on the performance of the different models [3, 13]. The covariates included were adult underweight proportion, alcohol (liters per capita), diabetes (fasting plasma glucose in mmol/L), education (years per capita), Healthcare Access and Quality [HAQ] Index, lag-distributed income (LDI), indoor air pollution, outdoor air pollution, population density (people per km2), smoking prevalence, cigarettes per capita, TB strain prevalence-weighted transmission risk, socio-demographic Index (SDI), prevalence of active TB, prevalence of latent TB infection, and a summary exposure variable scalar reflecting the average exposure to all of the risk factors [13]. The ensemble of CODEm models that performed best on out-of-sample predictive validity tests was selected [3, 10, 13]. HIV–TB deaths were estimated using a population attributable fraction approach taking into account baseline risk [10, 13]. In addition, GBD 2017 study addressed and adjusted the estimates of TB deaths in children for potential misclassification of TB deaths as pneumonia deaths in locations with high TB burden, such as Brazil [13]. A detailed description of modeling approaches to estimate TB deaths among HIV-negative and HIV-negative individuals been reported elsewhere [3, 13].

For estimating non-fatal TB burden, GBD 2017 study used all available data sources, including annual case notifications, prevalence surveys, population-based tuberculin surveys, and estimated cause-specific mortality of TB among HIV-positive and HIV-negative individuals [3, 10, 12]. GBD 2017 used DisMod-MR 2.1, a Bayesian meta-regression tool, to synthesize consistent non-fatal TB estimates by age, sex, year, and location [3, 10, 12]. This tool adjusts for variations in study methods between data sources and imposes consistency between data for different parameters [12]. To distinguish HIV-TB from all forms of TB, GBD study applied the proportions of HIV-TB cases among all TB cases estimated from a mixed-effects regression to TB incident and prevalent cases [10, 12]. The detailed description of the TB non-fatal modeling and estimation have been reported elsewhere [3, 10, 12]. In Brazil, the main sources of TB morbidity data used in GBD 2017 were the Notifiable Disease Information System (*Sistema de Informação de Agravos de Notificação*—*SINAN* in Portuguese) and notification data from the WHO’s global TB database, in addition to published national and subnational TB prevalence surveys [12, 18, 20, 21]. The TB morbidity and mortality data input sources used in GBD 2017 for Brazil is available at http://ghdx.healthdata.org/gbd-2017/data-input-sources.

### DALY calculation

In this study, DALYs and its components YLLs and YLDs were used to assess the fatal and non-fatal TB burden in Brazil. Additional details of DALY estimation methods have been described in the GBD publications [4]. YLLs were estimated by multiplying the number of TB deaths in each age by the GBD standard life expectancy at the age of death [4, 13]. In GBD 2017, the standard life expectancy at birth is 87.9 years, based on the lowest death rates for each age observed in countries with a population greater than 5 million [13]. YLDs were estimated by multiplying the prevalence of each sequelae or combination of sequelae related to TB by its disability weights, and then aggregating the estimates for all sequelae to the cause level [4, 12]. Disability weights quantify the relative severity of the sequelae on a scale from 0 (perfect health) to 1 (equivalent to death) and were derived from population-based surveys and an open web-based survey using pairwise comparison methods [4, 12]. Finally, DALYs were computed as the sum of YLLs and YLDs for each location, age group, sex, and year.

### Risk factor estimation

GBD 2017 used comparative risk assessment approaches to calculate the proportion of DALYs attributable to risk factors as a counterfactual to the hypothetical situation that populations had been exposed to a theoretical minimum level of exposure in the past [14]. The inclusion of a risk–outcome pair was based on the evidence of convincing or probable causal relationship between the risk and the outcome [14]. For TB among HIV-negative individuals, estimates were made of three risk factors (high fasting plasma glucose [HFPG], alcohol use, and smoking) because of evidence of their causal relationship with the risk of TB [10, 14]. To date, GBD has not quantified the contribution of some risk factors (e.g., indoor air pollution and malnutrition) that have been hypothesized to have a strong link with TB, because of insufficient evidence of a causal relationship [10]. Estimates of attributable DALYs were computed by multiplying DALYs for the outcome by the population attributable fraction for the risk-outcome pair for a given age, sex, location, and year [14].

Results are reported in absolute numbers and age-standardized rates (per 100,000 inhabitants) of DALYS, YLLs, and YLDs by sex, age group, year, and location with their respective 95% uncertainty intervals (UIs) based on the 25th and 975th values of the ordered 1000 draws of the uncertainty distribution [4, 12, 13].

We report positive and negative percentage changes to show increasing and decreasing variations between 1990 and 2017, respectively.

### Ethical considerations

This study was based on data which are publicly available and without nominal identification of individual data. The Project “Global Burden of Diseases—GBD in Brasil” was approved by the Research Ethics Committee of the Federal University of Minas Gerais (*Universidade Federal de Minas Gerais*—*UFMG*), Belo Horizonte, Brazil (protocol number 62803316.7.0000.5149).

## Results

### Levels and trends of national TB burden

In 2017, the number of DALYs due to TB (HIV-negative and HIV-positive combined) in Brazil was 284,323 (95% UI: 240,269–349,265) DALYs in Brazil (Table 1), accounting for 0.5% of national all-cause DALYs (about 60.5 million DALYs). Among HIV-negative individuals, the number of DALYs was 196,366 (95% UI: 189,645–202,394) (Additional file 1: Table S1), while 87,957 DALYs (95% UI: 50,624–146,870) were estimated among HIV-positive individuals (Additional file 2: Table S1). The number of DALYs due to TB at the national level decreased by 47.0% as compared to 1990 (535,982 DALYs [95% UI: 469,189–604,253]) (Table 1). The age-standardized DALY rates declined by 68.5% from 391.68 DALYs/100,000 inhabitants (95% UI: 347.64–436.53) in 1990 to 123.53 DALYs/100,000 inhabitants (95% UI: 104.30–151.83) in 2017 (Table 1). The same pattern of decline trend between 1990 and 2017 was observed for the number and age-standardized rates of DALYs due to TB both among HIV-negative (Additional file 1: Table S1) and HIV-positive individuals (Additional file 2: Table S1). YLLs were the main component of total DALYs due to TB over the period.

**Table 1:** Number of DALYs and age-standardized DALY rates (per 100,000 inhabitants) from tuberculosis in Brazil and states in 1990 and 2017, with absolute percentage change between 1990 and 2017

Region/State	Number of DALYs (95% UI)			Age-standardized DALY rates (per 100.000) (95% UI)		
	1990	2017	% Change 1990–2017	1990	2017	% Change 1990–2017
Brazil	535, 981.9 (469,188.8-604,252.6)	284,323.4 (240,268.9-349,264.8)	-47.0	391.68 (347.64-436.53)	123.53 (104.30-151.83)	-68.5
*North*						
Acre	1,977.7 (1,722.4-2,208.0)	1,434.6 (1,165.6-1,824.0)	-27.5	561.46 (505.23-610.72)	178.30 (146.75-223.63)	-68.2
Amapá	565.0 (482.0-655.5)	942.9 (765.3-1,185.8)	66.9	272.23 (240.04-309.76)	126.63 (104.77-156.04)	-53.5
Amazonas	8,866.1 (7,692.3-9,917.7)	8,600.9 (6,762.4-11,521.5)	-3.0	531.36 (476.21-576.02)	228.32 (183.40-299.05)	-57.0
Rondônia	3,477.5 (2,925.8-4,006.1)	1,940.5 (1,513.1-2,557.1)	-44.2	401.91 (348.83-453.63)	107.95 (84.68-140.64)	-73.1
Roraima	709.8 (567.3-883.6)	696.6 (535.3-936.2)	-1.9	401.28 (336.58-482.07)	132.47 (103.72-174.36)	-67.0
Pará	18,987.4 (15,911.5-21,876.8)	13,829.6 (11,134.5-17,982.8)	-27.2	445.16 (388.33-495.99)	163.64 (133.60-209.64)	-63.2
Tocantins	1,633.6 (1,127.4-2,145.0)	1,013.4 (802.0-1,309.7)	-38.0	197.50 (145.81-249.15)	62.85 (49.98-80.77)	-68.2
*Northeast*						
Alagoas	12,473.6 (9,969.3-15,064.0)	5,046.4 (4,074.4-6,567.5)	-59.5	491.13 (409.32-569.00)	142.90 (115.66-185.16)	-70.9
Bahia	55,857.5 (47,208.8-64,186.8)	26,466.2 (21,807.4-33,674.3)	-52.6	505.98 (442.08-567.77)	162.47 (133.75-206.93)	-67.9
Ceará	26,918.9 (21,213.1-32,988.2)	14,256.9 (11,496.0-18,425.0)	-47.0	424.39 (346.17-501.76)	141.58 (114.20-182.95)	-66.6
Maranhão	26,251.2 (21,977.0-31,624.5)	11,257.0 (9,070.6-14,604.5)	-57.1	572.74 (485.49-681.43)	151.37 (123.61-193.69)	-73.6
Paraíba	9,098.6 (7,572.5-10,663.2)	4,779.6 (3,790.8-6,206.6)	-47.5	307.20 (261.97-353.76)	106.80 (84.47-138.80)	-65.2
Pernambuco	46,819.1 (38,723.0-54,060.2)	24,877.4 (20,157.9-32,231.1)	-46.9	673.34 (578.18-761.26)	237.33 (192.75-307.14)	-64.8
Piauí	7,458.1 (5,805.0-9,343.2)	4,334.8 (3,489.1-5,625.6)	-41.9	313.95 (253.71-384.98)	118.29 (95.18-153.32)	-62.3
Rio Grande do Norte	7,403.8 (5,962.7-8,885.1)	4,655.2 (3,811.4-5,903.2)	-37.1	308.99 (259.11-362.18)	121.79 (99.47-154.40)	-60.6
Sergipe	4,402.6 (3,629.0-5,176.0)	2,765.5 (2,234.3-3,546.2)	-37.2	320.87 (272.12-367.69)	113.64 (92.14-145.17)	-64.6
*Southeast*						
Espírito Santo	5,931.2 (5,029.9-6,956.4)	3,756.4 (3,007.6-4,863.2)	-36.7	257.87 (224.12-295.89)	86.90 (69.44-112.98)	-66.3
Minas Gerais	30,730.1 (25,901.0-36,521.4)	16,312.3 (13,450.6-20,883.5)	-46.9	214.73 (185.76-249.87)	67.47 (55.19-87.06)	-68.6
Rio de Janeiro	91,401.2 (77,541.9-100,488.3)	48,986.9 (38,307.4-66,306.0)	-46.4	706.28 (603.15-774.83)	244.48 (189.72-333.17)	-65.4
São Paulo	11,6276.3 (94,073.8-146,075.9)	49,176.3 (40,012.2-63,008.1)	-57.7	373.85 (307.23-462.04)	94.65 (76.85-121.54)	-74.7
*South*						
Paraná	14,866.1 (12,496.7-17,780.1)	8,163.6 (6,534.2-10,573.5)	-45.1	199.29 (172.07-232.53)	64.34 (51.23-83.88)	-67.7
Rio Grande do Sul	22,239.8 (19,027.2-26,389.5)	15,286.4 (11,678.9-20,841.4)	-31.3	253.50 (219.01-297.90)	116.68 (88.59-160.20)	-54.0
Santa Catarina	5,222.8 (4,113.3-6,637.5)	3,400.1 (2,677.5-4,568.0)	-34.9	130.76 (106.43-161.78)	42.71 (33.44-57.98)	-67.3
*Central-West*						
Distrito Federal	1,733.4 (1,391.2-2,152.8)	1,028.2 (798.5-1,354.0)	-40.7	128.29 (108.06-152.81)	32.73 (25.66-42.87)	-74.5
Goiás	4,964.4 (3,940.3-6,274.2)	3,687.8 (2,997.5-4,717.5)	-25.7	140.63 (116.55-171.75)	49.51 (40.17-63.32)	-64.8
Mato Grosso	5,714.4 (4,824.4-6,818.8)	4,382.6 (3,484.5-5,793.1)	-23.3	342.34 (295.97-397.75)	118.24 (94.72-154.88)	-65.5
Mato Grosso do Sul	4,001.8 (3,263.5-4,830.3)	3,245.4 (2,590.0-4,273.9)	-18.9	262.48 (221.50-309.31)	108.22 (86.47-141.87)	-58.8

*DALYs* disability-adjusted life-years, *95*% *UI* 95% uncertainty interval

Like DALYs, the age-standardized rates of YLLs and YLDs due to TB at the national level decreased between 1990 and 2017 (Tables 2 and 3; Additional file 1: Tables S2 and S3; Additional file 2: Tables S2 and S3). However, higher declines were observed for age-standardized YLL rates compared to age-standardized YLD rates (Tables 2 and 3; Additional file 1: Tables S2 and S3; Additional file 2: Tables S2 and S3).

**Table 2:** Number of YLLs and age-standardized YLL rates (per 100,000 inhabitants) from tuberculosis in Brazil and states in 1990 and 2017, with absolute percentage change between 1990 and 2017

Region/State	Number of YLLs (95% UI)			Age-standardized YLL rates (per 100.000) (95% UI)		
	1990	2017	% Change 1990–2017	1990	2017	% Change 1990–2017
Brazil	526,951.1 (461,029.3-595,085.3)	273,637.3 (230,441.0-338,133.5)	-48.1	385.03 (341.38-429.64)	118.88 (100.03-146.93)	-69.1
*North*						
Acre	1,946.1 (1,691.4-2,174.0)	1,376.4 (1,105.2-1,761.3)	-29.3	551.92 (495.92-601.19)	171.46 (139.43-215.56)	-68.9
Amapá	548.3 (466.7-639.3)	896.3 (725.1-1,135.7)	63.4	264.49 (232.65-301.77)	120.71 (99.35-149.93)	-54.4
Amazonas	8,697.6 (7,523.3-9,747.0)	8,224.3 (6,407.4-11,085.7)	-5.4	521.16 (466.24-565.99)	218.72 (174.38-288.54)	-58.0
Rondônia	3,422.5 (2,874.9-3,944.0)	1,875.4 (1,445.9-2,490.1)	-45.2	395.94 (342.91-447.47)	104.39 (81.20-136.85)	-73.6
Roraima	696.7 (554.5-871.4)	668.7 (509.5-905.7)	-4.0	393.96 (329.15-474.02)	127.35 (98.78-168.74)	-67.7
Pará	18,683.9 (15,643.5-21,594.6)	13,309.6 (10,637.2-17,463.0)	-28.8	437.56 (381.02-489.01)	157.71 (127.99-203.77)	-64.0
Tocantins	1,601.1 (1,098.8-2,117.2)	961.1 (752.7-1,254.2)	-40.0	193.12 (140.81-244.76)	59.65 (46.93-77.44)	-69.1
*Northeast*						
Alagoas	12,351.9 (9,846.4-14,957.5)	4,898.0 (3,921.4-6,388.5)	-60.3	485.47 (403.35-563.47)	138.75 (111.59-180.13)	-71.4
Bahia	55,177.0 (46,505.1-63,496.7)	25,667.5 (21,009.7-32,883.7)	-53.5	499.30 (434.80-559.67)	157.60 (129.04-202.09)	-68.4
Ceará	26,572.6 (20,886.7-32,615.2)	13,729.4 (11,010.1-17,866.7)	-48.3	418.18 (340.69-494.13)	136.39 (109.42-177.35)	-67.4
Maranhão	25,964.0 (21,629.1-31,369.9)	10,873.4 (8,675.4-14,189.7)	-58.1	565.59 (476.74-676.26)	146.29 (118.44-188.40)	-74.1
Paraíba	8,953.4 (7,424.6-10,506.2)	4,603.9 (3,638.6-6,027.5)	-48.6	302.04 (256.98-348.70)	102.86 (81.10-134.49)	-65.9
Pernambuco	46,333.6 (38,236.4-53,583.3)	24,240.8 (19,532.0-31,579.9)	-47.7	665.73 (569.51-751.96)	231.23 (186.61-300.69)	-65.3
Piauí	7,339.4 (5,693.8-9,226.8)	4,170.2 (3,339.7-5,452.9)	-43.2	308.52 (248.91-378.82)	113.81 (91.29-148.67)	-63.1
Rio Grande do Norte	7,297.0 (5,873.9-8,769.3)	4,487.7 (3,646.0-5,728.2)	-38.5	304.01 (254.38-356.30)	117.43 (95.34-149.83)	-61.4
Sergipe	4,336.0 (3,558.1-5,111.3)	2,670.1 (2,148.7-3,437.8)	-38.4	315.66 (267.16-362.58)	109.77 (88.65-140.88)	-65.2
*Southeast*						
Espírito Santo	5,805.2 (4,919.9-6,828.2)	3,607.3 (2,864.0-4,698.6)	-37.9	252.54 (218.92-290.66)	83.42 (66.10-109.28)	-67.0
Minas Gerais	29,934.8 (25,038.6-35,638.7)	15,460.5 (12,598.7-19,979.1)	-48.4	209.30 (180.11-244.27)	63.89 (51.72-83.31)	-69.5
Rio de Janeiro	90,324.6 (76,365.0-99,367.8)	47,633.3 (37,023.7-64,945.6)	-47.3	698.07 (594.28-765.66)	237.60 (183.46-326.24)	-66.0
São Paulo	113,956.5 (91,550.1-143,738.6)	47,034.2 (38,069.7-60,819.3)	-58.7	366.47 (299.76-455.00)	90.41 (72.87-117.13)	-75.3
*South*						
Paraná	14,448.2 (12,068.1-17,338.7)	7,778.0 (6,188.9-10,152.4)	-46.2	193.95 (166.45-227.03)	61.24 (48.51-80.54)	-68.4
Rio Grande do Sul	21,566.2 (18,365.6-25,640.6)	14,595.7 (11,011.8-20,098.8)	-32.3	246.07 (211.23-289.88)	111.28 (83.00-154.38)	-54.8
Santa Catarina	5,022.9 (3,916.5-6,402.6)	3,152.8 (2,448.6-4,294.5)	-37.2	126.06 (101.51-156.85)	39.55 (30.59-54.62)	-68.6
*Central-West*						
Distrito Federal	1,667.3 (1,328.1-2,079.9)	932.9 (713.1-1,250.6)	-44.0	123.74 (103.51-148.16)	29.74 (23.03-39.78)	-76.0
Goiás	4,818.2 (3,798.5-6,125.9)	3,493.7 (2,824.3-4,492.4)	-27.5	136.64 (112.41-167.86)	46.87 (37.87-60.48)	-65.7
Mato Grosso	5,591.3 (4,693.1-6,676.1)	4,189.9 (3,297.3-5,587.5)	-25.1	335.13 (288.48-390.16)	113.07 (89.62-149.53)	-66.3
Mato Grosso do Sul	3,894.5 (3,156.3-4,720.9)	3,106.0 (2,447.6-4,122.6)	-20.2	255.75 (213.89-302.08)	103.55 (81.67-136.82)	-59.5

*YLLs* years of life lost, *95*% *UI* 95% uncertainty interval

**Table 3:** Number of YLDs and age-standardized YLD rates (per 100,000 inhabitants) from tuberculosis in Brazil and states in 1990 and 2017, with absolute percentage change between 1990 and 2017

Region/State	Number of YLDs (95% UI)			Age-standardized YLD rates (per 100.000) (95% UI)		
	1990	2017	% Change 1990–2017	1990	2017	% Change 1990–2017
Brazil	9,030.9 (6,022.1-12,395.5)	10,686.2 (7,002.8-14,963.4)	18.3	6.64 (4.43-9.05)	4.65 (3.05-6.50)	-30.0
*North*						
Acre	31.6 (20.6-44.7)	58.1 (37.0-85.0)	83.7	9.54 (6.36-13.25)	6.83 (4.38-9.85)	-28.4
Amapá	16.7 (10.9-24.2)	46.6 (29.8-67.0)	179.1	7.74 (5.15-10.90)	5.91 (3.86-8.38)	-23.6
Amazonas	168.5 (107.7-238.3)	376.6 (237.6-551.8)	123.4	10.20 (6.71-14.26)	9.60 (6.17-13.88)	-5.9
Rondônia	54.9 (34.5-79.1)	65.1 (40.5-96.5)	18.5	5.97 (3.82-8.39)	3.55 (2.23-5.21)	-40.5
Roraima	13.0 (8.5-18.8)	27.9 (17.2-40.5)	113.8	7.32 (4.80-10.43)	5.11 (3.21-7.32)	-30.1
Pará	303.5 (193.9-437.1)	520.0 (329.5-764.2)	71.3	7.61 (4.97-10.82)	5.92 (3.78-8.70)	-22.1
Tocantins	32.5 (20.9-46.9)	52.3 (33.3-75.9)	60.8	4.38 (2.81-6.21)	3.21 (2.03-4.64)	-26.8
*Northeast*						
Alagoas	121.7 (79.4-173.9)	148.4 (92.8-219.4)	21.9	5.66 (3.74-8.09)	4.16 (2.61-6.13)	-26.5
Bahia	680.5 (441.3-966.4)	798.6 (502.5-1,165.1)	17.4	6.69 (4.40-9.33)	4.87 (3.06-7.10)	-27.2
Ceará	346.3 (228.2-495.2)	527.5 (338.0-758.9)	52.4	6.21 (4.09-8.82)	5.19 (3.35-7.44)	-16.5
Maranhão	287.1 (184.8-407.3)	383.6 (237.9-559.8)	33.6	7.16 (4.63-10.14)	5.08 (3.20-7.37)	-29.1
Paraíba	145.2 (94.2-208.3)	175.7 (111.4-257.3)	21.0	5.16 (3.33-7.40)	3.93 (2.49-5.74)	-23.8
Pernambuco	485.5 (316.6-687.5)	636.5 (406.3-926.7)	31.1	7.61 (5.02-10.71)	6.10 (3.91-8.89)	-19.8
Piauí	118.7 (77.2-167.9)	164.6 (103.0-237.8)	38.7	5.43 (3.52-7.67)	4.48 (2.80-6.46)	-17.4
Rio Grande do Norte	106.8 (69.4-151.5)	167.5 (105.6-248.1)	56.9	4.98 (3.26-7.10)	4.36 (2.75-6.42)	-12.5
Sergipe	66.6 (43.3-94.5)	95.4 (60.2-137.2)	43.2	5.20 (3.37-7.30)	3.87 (2.44-5.56)	-25.6
*Southeast*						
Espírito Santo	126.0 (80.5-181.8)	149.1 (94.2-213.1)	18.3	5.33 (3.48-7.62)	3.48 (2.21-4.98)	-34.8
Minas Gerais	795.3 (505.7-1,138.7)	851.8 (541.2-1,239.0)	7.1	5.43 (3.49-7.64)	3.58 (2.27-5.190	-34.0
Rio de Janeiro	1,076.5 (688.3-1,538.1)	1,353.5 (864.3-1,991.1)	25.7	8.21 (5.31-11.65)	6.88 (4.40-10.11)	-16.1
São Paulo	2,319.8 (1,434.3-3,299.3)	2,142.1 (1,374.5-3,108.4)	-7.7	7.38 (4.69-10.39)	4.24 (2.72-6.14)	-42.6
*South*						
Paraná	417.9 (265.7-604.1)	385.6 (240.0-553.7)	-7.7	5.34 (3.45-7.66)	3.09 (1.94-4.42)	-42.1
Rio Grande do Sul	673.6 (436.2-958.3)	690.7 (441.8-1,008.3)	2.5	7.43 (4.93-10.59)	5.40 (3.44-7.94)	-27.4
Santa Catarina	199.9 (126.7-287.3)	247.3 (155.1-364.0)	23.7	4.70 (3.02-6.70)	3.17 (1.99-4.68)	-32.6
*Central-West*						
Distrito Federal	66.1 (43.0-95.4)	95.3 (59.3-141.2)	44.3	4.55 (3.05-6.48)	3.00 (1.88-4.39)	-34.1
Goiás	146.2 (94.0-210.3)	194.1 (121.8-284.4)	32.7	3.99 (2.61-5.70)	2.63 (1.67-3.84)	-33.9
Mato Grosso	123.1 (79.3-174.6)	192.7 (119.9-285.1)	56.6	7.20 (4.64-10.09)	5.17 (3.26-7.61)	-28.2
Mato Grosso do Sul	107.2 (67.0-151.6)	139.4 (88.4-203.3)	30.0	6.73 (4.34-9.34)	4.67 (2.99-6.79)	-30.6

*YLDs* years lived with disability, *95*% *UI* 95% uncertainty interval

### TB burden by age and sex

**Fig. 1:**
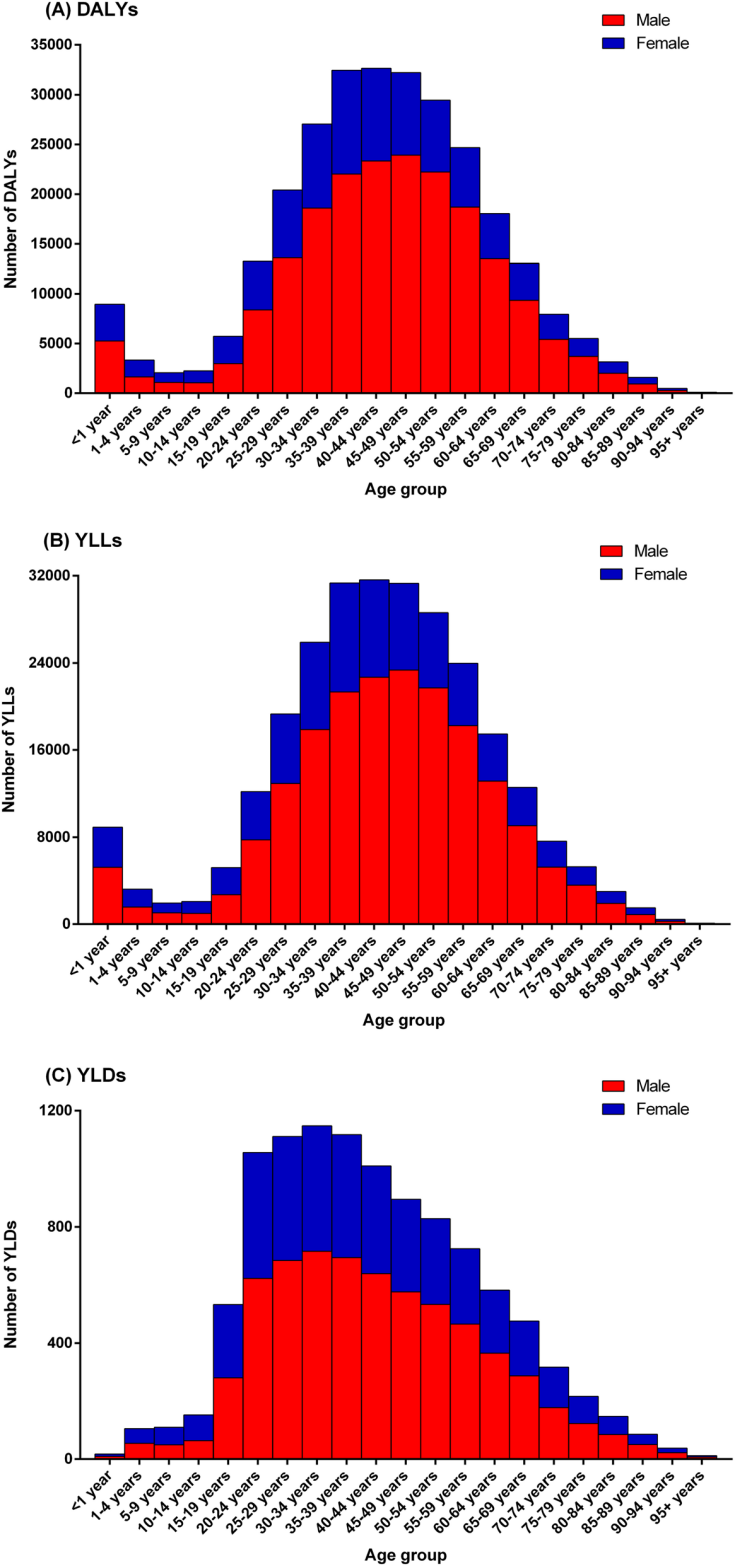
Absolute number of age- and sex-specific **a** DALYs, **b** YLLs, and **c** YLDs from tuberculosis in Brazil, 2017. *DALYs* disability-adjusted life-years, *YLLs* years of life lost, *YLDs* years lived with disability

In 2017, the age-standardized DALY rate (HIV-negative and HIV-positive combined) among males (177.75 DALYs/100,000 inhabitants [95% UI: 151.51–215.89]) was 2.4 times higher than that among females (74.19 DALYs/100,000 inhabitants [95% UI: 60.43–94.67]). The number of age-specific DALYs for both sexes combined was highest in the age groups 30–54 years (with a peak in the aged 40–44 years) (Fig. 1a). Among HIV-negative individuals, the number of DALYs was highest among people aged 50–54 years (Additional file 3: Figure S1-A), while among HIV-positive individuals, in the aged 30–35 years (Additional file 4: Figure S2-A).

**Fig. 2:**
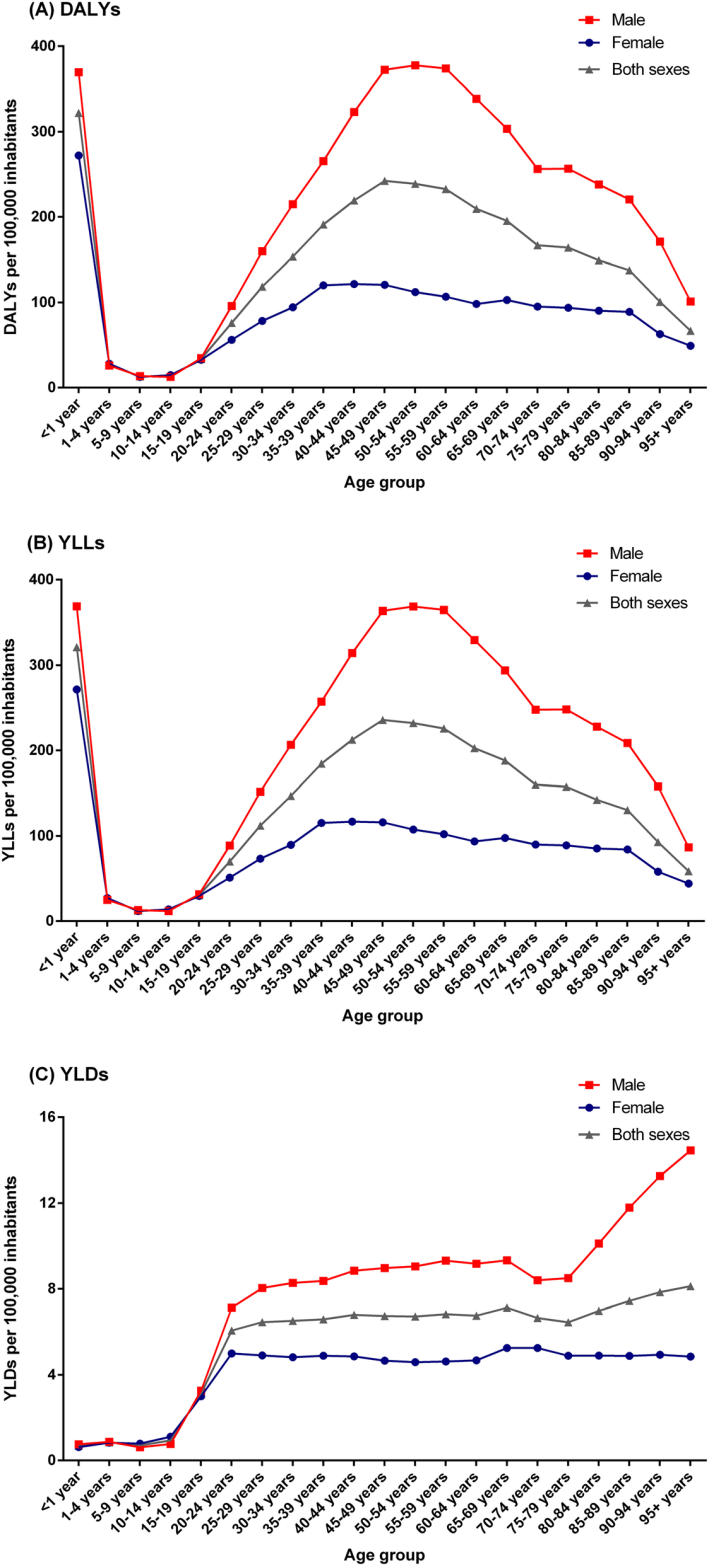
Age- and sex-specific rates (per 100,000 inhabitants) of **a** DALYs, **b** YLLs, and **c** YLDs from tuberculosis in Brazil, 2017. *DALYs* disability-adjusted life-years, *YLLs* years of life lost, *YLDs* years lived with disability

The age-specific DALY rates for both sexes combined were highest among children under 1 year (321.98 DALYs/100,000 inhabitants [95% UI: 232.50–443.29]) and in the age groups 45–59 years (> 230 DALYs/100,000 inhabitants) (Fig. 2a). The similar pattern was observed for both HIV-negative (Additional file 5: Figure S3-A) and HIV-positive individuals (Additional file 6: Figure S4-A), with the highest age-specific DALY rates for both sexes combined observed among children under 1 year. For males, the age-specific DALY rates were highest in the aged 50–54 years (377.61 DALYs/100,000 inhabitants [95% UI: 321.16–456.12]), while for females were observed in children under 1 year (272.27 DALYs/100,000 inhabitants [95% UI: 195.50–375.27]) (Fig. 2a). Among HIV-positive individuals, the DALY rates for males were highest in children under 1 year (Additional file 6: Figure S4-A).

Similar to DALYs, the age-specific rates of YLLs and YLDs due to TB were highest in males. The age-specific YLL rates for both sexes combined were highest among children under 1 year (321.29 YLLs/100,000 inhabitants [95% UI: 231.76–442.44) (Fig. 2a). Among HIV-negative individuals, the highest age-specific YLL rates for both sexes combined were observed among children under 1 year and in the aged 55–59 years (Additional file 5: Figure S3-B), while among HIV-positive individuals, the highest YLL rate was observed among children under 1 year (Additional file 6: Figure S4-B). For males, the age-specific YLL rates were highest among children under 1 year and in the age groups 45–59 years (> 360 YLLs/100,000 inhabitants), while for females were observed in children under 1 year (271.65 YLLs/100,000 inhabitants [95% UI: 194.60–374.58]) (Fig. 2b).

The age-specific YLD rates due to TB for both sexes combined were highest among those aged above 85 years, with a peak in the age group 95 years and older (8.13 YLDs/100,000 inhabitants [95% UI: 5.10–12.14]) (Fig. 2c). Among HIV-positive individuals, the age-specific YLD rates for both sexes combined were highest in the age group 35–39 years (Additional file 6: Figure S4-C). For males, the highest age-specific YLD rate was observed in the age group 95 years and older (14.47 YLDs/100,000 inhabitants [95% UI: 8.98–21.70]), while for females was observed in the age group 65–74 years (> 5.25 YLDs/100,000 inhabitants) (Fig. 2c).

### Regional variations in TB burden

**Fig. 3:**
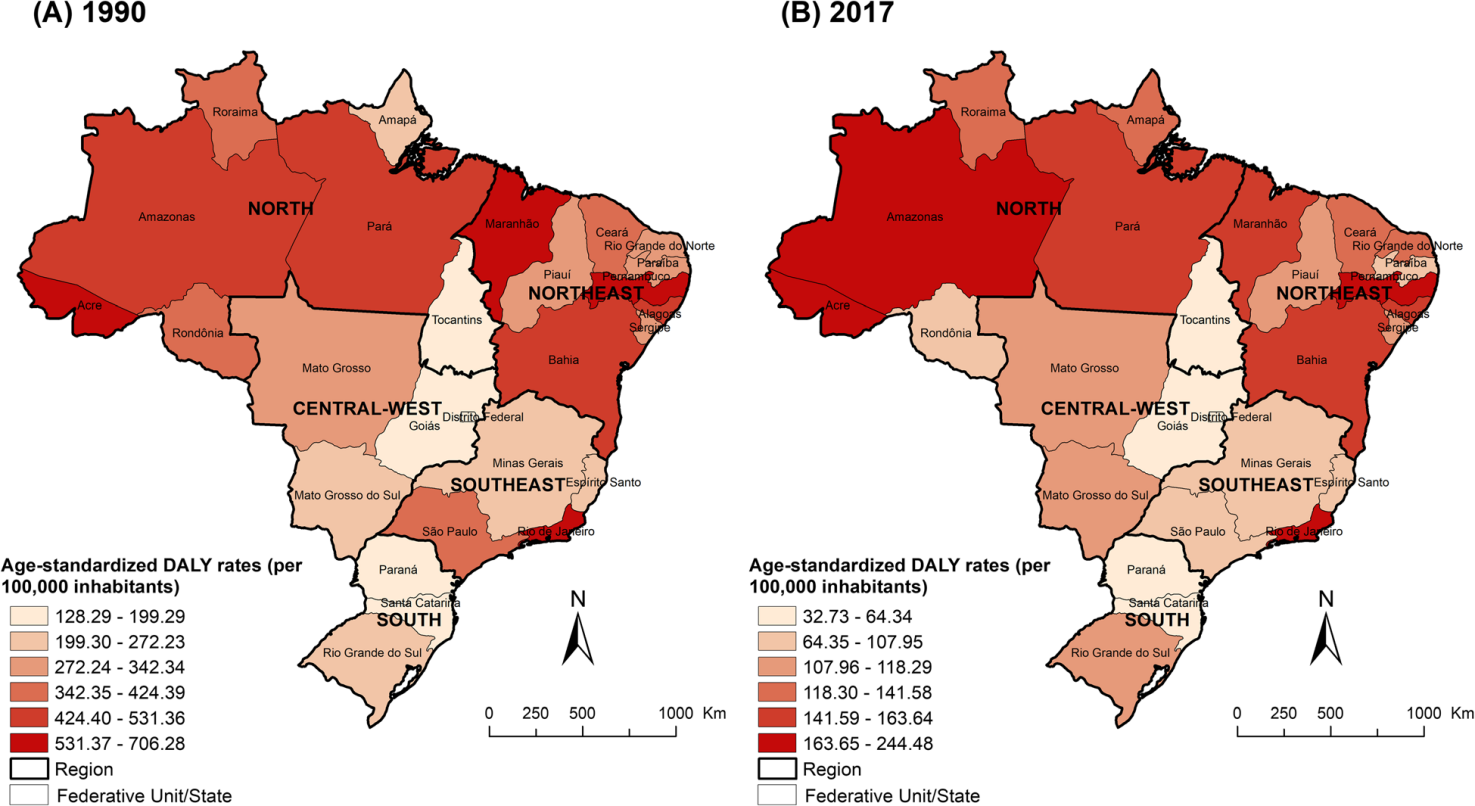
Age-standardized DALY rates (per 100,000 inhabitants) from tuberculosis by states in Brazil for 1990 and 2017. *DALY* disability-adjusted life-years

Table 1 presents the number of DALYs and age-standardized DALY rates due to TB (HIV-negative and HIV-positive individuals combined) by Brazilian states for 1990 and 2017. Estimates of DALYs due to TB among HIV-negative and HIV-positive individuals by Brazilian states are presented in Additional file 1: Table S1 and Additional file 2: Table S1, respectively. In 2017, the number of DALYs due to TB was highest in the states of São Paulo, Rio de Janeiro, and Bahia, whereas the age-standardized DALY rates (> 220 DALYs/100,000 inhabitants) were highest in the states of Rio de Janeiro, Pernambuco, and Amazonas (Fig. 3). A similar pattern was also observed for both HIV-negative (Additional file 7: Figure S5) and HIV-positive individuals (Additional file 8: Figure S6), with the highest age-standardized DALY rates observed in the same Brazilian states. The age-standardized DALY rates due to TB decreased for all 27 Brazilian states between 1990 and 2017, with the highest decreases observed in the states of São Paulo, Distrito Federal, Maranhão, Rondônia, and Alagoas (a relative reduction of 70% or more), while the smallest decreases were observed in the states of Amapá, Rio Grande do Sul, Amazonas, and Mato Grosso do Sul (Table 1).

The age-standardized YLL rates were highest in the states of Rio de Janeiro, Pernambuco, and Amazonas, with a declining trend of age-standardized YLL rates for all Brazilian states between 1990 and 2017 (Table 2). The age-standardized YLD rates were highest in the states of Amazonas, Rio de Janeiro, and Acre (Table 3). Age-standardized YLD rates decreased for all 27 Brazilian states between 1990 and 2017, with slower declines in age-standardized rates when compared to YLLs (Table 3). Among HIV-positive individuals, the age-standardized YLD rates were highest in the states of Amazonas, Rio de Janeiro, and Rio Grande do Sul, with an increasing trend of age-standardized YLD rates in several Brazilian states between 1990 and 2017 (Additional file 2: Table S3).

### TB burden attributable to risk factors

**Fig. 4:**
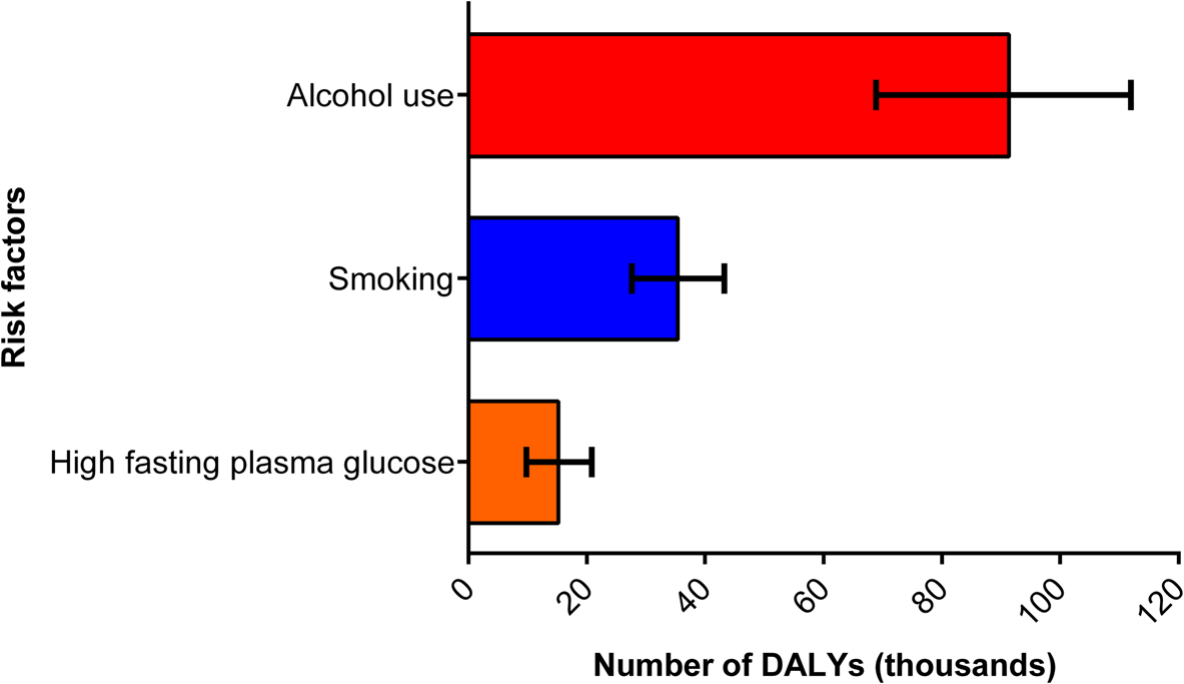
Number of tuberculosis DALYs (with 95% uncertainty intervals) attributable to alcohol use, smoking, and diabetes in Brazil, 2017. *DALYs* disability-adjusted life-years

In 2017, 59.0% (115,819 DALYs [95% UI: 97,688–131,549]) of total DALYs due to TB among HIV-negative individuals in Brazil were attributable to alcohol use, smoking, and diabetes, compared to 43.6% (173,820 DALYs [95% UI: 151,254–194,654]) of total DALYs in 1990. Alcohol use accounted for 47.5% of national DALYs due to TB in 2017, smoking for 17.9%, and diabetes for 7.7% (Fig. 4). The proportion of DALYs due to TB attributable to three risk factors decreased between 1990 and 2017, with a higher decline of DALYs attributable to smoking (percentage change: − 61.4%) than compared with those attributable to diabetes (− 27.4%) and alcohol use (− 19.6%).

## Discussion

This study is the first comprehensive national assessment of the levels and trends in fatal and non-fatal burden of TB in Brazil over 28 years (1990–2017). GBD 2017 findings showed a consistent decreasing trend of age-standardized DALY rates at the national level and all Brazilian states from 1990 to 2017, driven primarily by a faster decrease in the age-standardized YLL rates, the main component of total DALYs due to TB. The highest TB burden was observed among males, middle-aged adults and children under 1 year, and in highly endemic states for the disease in Brazil. HIV-TB comprised about 31% of DALYs due to TB and alcohol use was the leading risk factor for the attributable TB burden among HIV-negative individuals in 2017.

The high TB burden in males observed in the GBD 2017 study reflects the patterns of the disease in Brazil since most of the TB cases and deaths are recorded in this gender in the country [6, 22]. The predominance of fatal and non-fatal burden due to TB among males can be explained by the higher exposure to bacillus in work activities and the frequency of risk behaviors for the disease in this gender, such as higher consumption of alcoholic beverages, smoking, and drug use [10, 23]. Besides, the highlights was sex difference in TB risk due to health care behavior, such as lower self-care, lower demand for health care, and lower adherence and greater abandonment loss of follow-up to TB treatment among males [10, 24, 25]. Thus, an understanding of the sex distribution of TB cases and deaths has implications for TB control programmes concerning targeting of interventions to high-risk groups [10].

The highest number of DALYs and age-specific DALY rates for TB observed among middle-aged adults (35–64 years) corroborates with age-specific patterns of disease in the country [22]. The highest concentration of TB burden in the age group of greater economic productivity generates a socio-economic impact on patients, their family, and society, causing more poverty and social exclusion [8]. Also, it is important to highlight the high DALY rate due to TB in children under 1 year of age, primarily due to the high rates of fatal burden estimated in this age group [13]. The high burden of premature TB deaths in this age group observed in GBD 2017, different of the previous GBD cycles, might be explained by the changes in the estimation process with adjustments and corrections for potential misclassification of TB deaths in children [3, 13]. In locations with high TB burden, such as Brazil, TB mortality in children may be underestimated because many children deaths caused by TB may be erroneously attributed to more common diseases in this age, such as pneumonia, HIV/AIDS, and meningitis [26–29]. GBD 2017 estimates identify a prime opportunity to address an under-recognized and preventable cause of premature death in children under 1 year, and should motivate the ongoing development of better methods for detecting or preventing cases of pediatric TB [3]. Although TB is curable, diagnostic methods for TB have poor performance due to difficulties obtaining samples and paucibacillary form in children [28]. Thus, the dramatic reduction of pediatric TB burden requires better diagnostic technology, along with significant advances in treatment and vaccination [29]. However, in the meantime, more consistent preventive treatment for children exposed to TB and more persistent assessment of potential pediatric TB cases can take too long before leading to a reduction in the mortality burden [29]. Efforts for the prevention, diagnosis, and treatment of childhood TB should consider the peculiarities of the disease in this age group, ensuring the early identification and treatment of active and latent TB infection (LTBI) and using more sensitive and less invasive methods for the diagnosis of extrapulmonary and paucibacillary pulmonary TB [30].

We observed regional variations in the TB burden among Brazilian states. Except for the state of Rio de Janeiro (Southeast region), the highest age-standardized DALY rates in 2017 were observed in states of the North and Northeast regions. The findings of TB burden by Brazilian states corroborate with the observed patterns of regional TB morbidity and mortality in the country [22, 31]. The highest mortality and incidence rates of TB were observed in the states of Rio de Janeiro, Pernambuco, and Amazonas [6, 22]. This pattern of TB distribution can be explained in part by the occurrence of cases and deaths that are concentrated mainly in peripheral urban areas, with clusters of patients living in extreme poverty and inadequate housing, facilitating the transmission of the disease, a scenario mainly observed in the states of Rio de Janeiro and Pernambuco [6, 32, 33]. In the states of North region, in addition to the close relationship of TB burden with precarious living conditions and densely populated areas in some cities in this region, stands out the occurrence of TB in indigenous population, a recognized vulnerable group for TB and other major endemic diseases in the Amazon region, which may influence in part the TB burden in some areas with a high proportion of this population in the region [34].

The steady decline of age-standardized DALY rates due to TB at the national level from 1990 to 2017 corroborates the observed patterns of decreasing trends in TB mortality and incidence in the last decades [6, 22]. Substantial progress has been made in reducing TB incidence and mortality in Brazil, with the country meeting the Millennium Development Goals (MDGs) targets for TB control by 2015 [31, 35, 36]. This reduction can be explained by the progress in the access and quality of diagnostic and treatment services provided to patients with TB combined with improvements in the living conditions of the Brazilian population [37]. However, despite the general progress in the reduction of TB mortality and morbidity, the disease burden and its control still remain a challenge [6, 31]. The progress is not homogeneous among states and municipalities, and is insufficient to reach the agreed global targets for End TB Strategy [37]. In this sense, compliance with the goals established by WHO and SDGs and agreed by the Brazilian Ministry of Health will be difficult to achieve without major and profound changes in the access, coverage, and quality of the health care provided, adherence to a social protection agenda, and changes in the regional economic and political context [30, 36]. Thus, efforts should be made to strengthen TB surveillance and control actions in Brazil through systematic strategies that promote and improve access to prevention, early diagnosis and timely treatment, and appropriate follow-up of TB cases, seeking to reduce TB cases and deaths in the country [1, 38]. Along with intensifying the active search for TB cases, further efforts should be made to prioritize the systematic screening and control of household contacts of bacteriologically confirmed pulmonary TB cases, which allows the identification of people with LTBI, enabling receiving TB preventive treatment to reduce the risk of progression to active TB, in addition to diagnose early cases of active disease and start treatment timely [1, 38]. Since TB is an infectious disease that is strongly related to social inequality in Brazil, this reinforces the need to intensify multisectoral actions addressing the economic and social determinants of TB infection and disease, giving special attention to poor and vulnerable populations and communities especially at risk [1, 39]. The implementation and expansion of public initiatives for social protection and poverty alleviation, such as the *Bolsa Família* Programme, the national conditional cash transfer programme, can contribute in TB prevention and care and to favorable TB treatment outcomes among vulnerable populations in Brazil [39, 40].

The considerable proportion of estimates of DALYs due to TB among HIV-positive individuals reinforces the epidemiological relevance of TB-HIV co-infection as a comorbidity of significant impact on public health in Brazil [41, 42]. Currently, despite the improvements in the quality of management and care of TB in people living with HIV/AIDS and increased access to active antiretroviral therapy (ART) in Brazil, morbidity and mortality due to TB continues remains high among HIV-positive people in the country, impacting negatively the survival and quality of life of this population [43]. TB is the leading cause of death among infectious diseases defined in HIV/AIDS patients in the country [44, 45]. The two diseases (mainly TB) are mostly concentrated in areas of poverty where there are minimal resources for the diagnosis, treatment, and control of infection, and public health services do not meet the needs for control of both pathologies [41, 46]. The Brazilian Ministry of Health recommends that all patients with TB undergo HIV serological testing [45]. This procedure allows an early diagnosis of HIV infection, allowing, when indicated, the timely initiation of ART, which has a significant impact on the survival of these patients and resulting in a reduction in its morbidity and mortality [10, 45]. Despite this orientation, low rates of HIV screening in some Brazilian areas with poorer health service structure is observed, which increases the uncertainty about the real magnitude of TB-HIV co-infection and makes difficult its early diagnosis and timely ART initiation [41, 45, 47]. Furthermore, it is important to highlight the influence of social stigma and prejudice regarding the two diseases as negative implication in the timely diagnosis and adequate treatment of TB-HIV cases [48]. In this context, in order to overcome the challenges of reducing the burden of co-infection in the country, reinforces the need for greater integration between TB and HIV control programs and services, with a multidisciplinary approach and integral care, regarding the early identification of co-infection and timely use of ART and the TB treatment regimen, considering that these actions are mainly aimed in improving the quality of life and reducing the severe TB-HIV cases and deaths [10, 49].

The assessment of the contribution of potentially modifiable risk factors is also a crucial input into TB control policy [10]. Nationally, GBD 2017 estimated that alcohol use, smoking, and diabetes together accounted for about 60% of the proportion of DALYs due to TB among HIV-negative people in 2017. These risk factors could increase the risk of TB through suppression of the immune system, especially cell-mediated immunity [50–53]. Alcohol use, the leading risk factor observed in the study, causes a decline in immunity, malnutrition, and social fragility, being considered as an important risk factor for the development of TB, but it also influences the outcome of TB treatment and prognosis [10, 51, 53, 54]. Therefore, efforts to prevent these health-related risk factors, along with HIV, can have a substantial impact on the TB burden [3, 10].

The general limitations of the GBD 2017 approach and those for estimation of TB burden are described elsewhere [3, 4, 12–14, 16]. GBD specific limitations for Brazil, such as coverage, quality, and availability of epidemiological data used to estimate the disease burden, have also been detailed in previous publications [18, 19, 55]. For mortality data, although the *SIM* database has experienced substantial improvements and progress in coverage and quality of information on causes of death since 1990, mortality coverage and proportion of ill-defined causes presented variations among the Brazilian states, with higher proportion of underreporting of deaths and ill-defined causes of death in some states in the North and Northeast regions [18, 55, 56]. Furthermore, the underlying cause of death may have been coded as a complication or aggravation associated with TB [57]. Although the GBD study uses comparable and standardized methods processes for correction of underreporting of deaths and redistribution of garbage codes, the regional variations can substantially affect mortality estimates, which should be interpreted with caution for some Brazilian states [13, 19, 21].

For non-fatal data, despite improvements in the coverage and quality of epidemiological surveillance data for TB in the country (*SINAN* database) in recent years, specific limitations such as the underreporting or incorrect reporting of TB cases and completeness of the variables can occur in locations with precarious structure of health services [56, 58]. This can present critical challenges to the production of accurate estimates of non-fatal TB burden, particularly at the state level [3, 10]. Thus, the TB estimates for some Brazilian locations with limited high-quality data are reflected in the wide uncertainty intervals [3, 10].

Despite these limitations, the levels and trends of TB burden are consistent with the epidemiological patterns of the disease in the country. In addition, the GBD estimates are relevant to demonstrate the importance of TB as a cause of years of healthy life lost due to premature death and disability in Brazil and its states, besides to provide an up-to-date comparative assessment of TB burden with other countries or regions.

## Conclusions

GBD 2017 results show that, despite general progress in reduction of TB burden in Brazil during the 28-year study period (1990–2017), the disease is still an important preventable and treatable cause of health loss due to premature death and disability. The TB burden was higher in males, children under 1 year and middle-aged adults (aged 40–64 years), and in the Brazilian states with the highest incidence/mortality rates. The findings reinforce the importance of strengthening control and surveillance strategies in Brazil through integrated and multisectoral actions that enable the access to prevention, early diagnosis, and timely and adequate treatment of TB, with emphasis on high-risk groups and populations most vulnerable to the disease.

## Supplementary information

**Additional file 1: Table S1.** Number of DALYs and age-standardized DALY rates (per 100,000 inhabitants) from tuberculosis among HIV-negative individuals in Brazil and states in 1990 and 2017, with absolute percentage change between 1990 and 2017. **Table S2.** Number of YLLs and age-standardized YLL rates (per 100,000 inhabitants) from tuberculosis among HIV-negative individuals in Brazil and states in 1990 and 2017, with absolute percentage change between 1990 and 2017. **Table S3.** Number of YLDs and age-standardized YLD rates (per 100,000 inhabitants) from tuberculosis among HIV-negative individuals in Brazil and states in 1990 and 2017, with absolute percentage change between 1990 and 2017. DALYs = disability-adjusted life-years. YLLs = years of life lost. YLDs = years lived with disability. 95% UI = 95% uncertainty interval.

**Additional file 2: Table S1.** Number of DALYs and age-standardized DALY rates (per 100,000 inhabitants) from tuberculosis among HIV-positive individuals in Brazil and states in 1990 and 2017, with absolute percentage change between 1990 and 2017. **Table S2.** Number of YLLs and age-standardized YLL rates (per 100,000 inhabitants) from tuberculosis among HIV-positive individuals in Brazil and states in 1990 and 2017, with absolute percentage change between 1990 and 2017. **Table S3.** Number of YLDs and age-standardized YLD rates (per 100,000 inhabitants) from tuberculosis among HIV-positive individuals in Brazil and states in 1990 and 2017, with absolute percentage change between 1990 and 2017. DALYs = disability-adjusted life-years. YLLs = years of life lost. YLDs = years lived with disability. 95% UI = 95% uncertainty interval.

**Additional file 3: Figure S1.** Absolute number of age- and sex-specific (A) DALYs, (B) YLLs, and (C) YLDs from tuberculosis among HIV-negative individuals in Brazil, 2017. DALYs = disability-adjusted life-years; YLLs = years of life lost; YLDs = years lived with disability.

**Additional file 4: Figure S2.** Absolute number of age- and sex-specific (A) DALYs, (B) YLLs, and (C) YLDs from tuberculosis among HIV-positive individuals in Brazil, 2017. DALYs = disability-adjusted life-years; YLLs = years of life lost; YLDs = years lived with disability.

**Additional file 5: Figure S3.** Age- and sex-specific rates (per 100,000 inhabitants) of (A) DALYs, (B) YLLs, and (C) YLDs from tuberculosis among HIV-negative individuals in Brazil, 2017. DALYs = disability-adjusted life-years; YLLs = years of life lost; YLDs = years lived with disability.

**Additional file 6: Figure S4.** Age- and sex-specific rates (per 100,000 inhabitants) of (A) DALYs, (B) YLLs, and (C) YLDs from tuberculosis among HIV-positive individuals in Brazil, 2017. DALYs = disability-adjusted life-years; YLLs = years of life lost; YLDs = years lived with disability.

**Additional file 7: Figure S5.** Age-standardized DALY rates (per 100,000 inhabitants) from tuberculosis among HIV-negative individuals by states in Brazil for 1990 and 2017. DALY = disability-adjusted life-years.

**Additional file 8: Figure S6.** Age-standardized DALY rates (per 100,000 inhabitants) from tuberculosis among HIV-positive individuals by states in Brazil for 1990 and 2017. DALY = disability-adjusted life-years.

## Data Availability

GBD 2017 results by location (including Brazil and its 27 states) and year are publicly available through of online visualization tools: https://vizhub.healthdata.org/gbd-compare and http://ghdx.healthdata.org/gbd-results-tool.

## Abbreviations

ART: Antiretroviral therapy
CODEm: Cause of Death Ensemble model
DALYs: Disability-adjusted life years
ESF: *Estratégia de Saúde da Família*
GBD: Global burden of disease
HFPG: High fasting plasma glucose
ICD: International Classification of Diseases
IHME: Institute for Health Metrics and Evaluation
LTBI: Latent tuberculosis infection
MDGs: Millennium Development Goals
SDGs: Sustainable development goals
SIA/SUS: *Sistema de Informações Ambulatoriais do SUS*
SIH/SUS: *Sistema de Informações Hospitalares do Sistema Único de Saúde*
SIM: *Sistema de Informações sobre Mortalidade*; SINAN: *Sistema de Informação de Agravos de Notificação*
TB: Tuberculosis
UI: Uncertainty interval
WHO: World Health Organization
YLDs: Years lived with disability
YLLs: Years of life lost

## References

[1] World Health Organization (2018). Global tuberculosis report 2019.

[2] Houben RM (2016). Dodd PJ The global burden of latent tuberculosis infection: a re-estimation using mathematical modelling. PLoS Med.

[3] GBD Tuberculosis Collaborators (2018). Global, regional, and national burden of tuberculosis, 1990–2016: results from the Global Burden of Diseases, Injuries, and Risk Factors 2016 Study. Lancet Infect Dis.

[4] GBD 2017 DALYs and HALE Collaborators (2018). Global, regional, and national disability-adjusted life-years (DALYs) for 359 diseases and injuries and healthy life expectancy (HALE) for 195 countries and territories, 1990–2017: a systematic analysis for the Global Burden of Disease Study 2017. Lancet.

[5] Pelissari DM, Rocha MS, Bartholomay P, Sanchez MN, Duarte EC, Arakaki-Sanchez D (2018). Identifying socioeconomic, epidemiological and operational scenarios for tuberculosis control in Brazil: an ecological study. BMJ Open.

[6] Ministério da Saúde. Secretaria de Vigilância em Saúde. Tuberculose - Situação Epidemiológica da Tuberculose. http://saude.gov.br/saude-de-a-z/tuberculose#epidemiologia. Brasília: Ministério da Saúde; 2019. Accessed 20 Dec 2019.

[7] Vilela PNS, Schneider IJC, Traebert E, Traebert J (2018). Burden of tuberculosis trends in a Brazilian southern state. Rev Bras Epidemiol.

[8] Ferrer GC, da Silva RM, Ferrer KT, Traebert J (2014). The burden of disease due to tuberculosis in the state of Santa Catarina, Brazil. J Bras Pneumol.

[9] United Nations (2015). Sustainable Development Goals: 17 Goals to transform our world. 2015.

[10] GBD Tuberculosis Collaborators (2018). The global burden of tuberculosis: results from the Global Burden of Disease Study 2015. Lancet Infect Dis.

[11] Uplekar M, Weil D, Lonnroth K, Jaramillo E, Lienhardt C, Dias H (2015). WHO’s new end TB strategy. Lancet..

[12] GBD 2017 Disease and injury incidence and prevalence collaborators (2018). Global, regional, and national incidence, prevalence, and years lived with disability for 354 diseases and injuries for 195 countries and territories, 1990–2017: a systematic analysis for the Global Burden of Disease Study 2017. Lancet.

[13] GBD 2017 Causes of Death Collaborators (2018). Global, regional, and national age-sex-specific mortality for 282 causes of death in 195 countries and territories, 1980–2017: a systematic analysis for the Global Burden of Disease Study 2017. Lancet.

[14] GBD 2017 Risk Factor Collaborators (2018). Global, regional, and national comparative risk assessment of 84 behavioural, environmental and occupational, and metabolic risks or clusters of risks for 195 countries and territories, 1990–2017: a systematic analysis for the Global Burden of Disease Study 2017. Lancet.

[15] GBD 2016 Disease and injury incidence and prevalence collaborators (2017). Global, regional, and national incidence, prevalence, and years lived with disability for 328 diseases and injuries for 195 countries, 1990–2016: a systematic analysis for the Global Burden of Disease Study 2016. Lancet.

[16] GBD 2017 Mortality Collaborators (2018). Global, regional, and national age-sex-specific mortality and life expectancy, 1950–2017: a systematic analysis for the Global Burden of Disease Study 2017. Lancet.

[17] Instituto Brasileiro de Geografia e Estatística. Estimativas da População – 2019. Brasília: Instituto Brasileiro de Geografia e Estatística; 2019. https://www.ibge.gov.br/estatisticas/sociais/populacao/9103-estimativas-de-populacao.html?=&t=resultados. Accessed 22 Dec 2019.

[18] GBD 2016 Brazil Collaborators (2018). Burden of disease in Brazil, 1990–2016: a systematic subnational analysis for the Global Burden of Disease Study 2016. Lancet.

[19] França EB, Passos VMA, Malta DC, Duncan BB, Ribeiro ALP, Guimarães MDC (2017). Cause-specific mortality for 249 causes in Brazil and states during 1990–2015: a systematic analysis for the global burden of disease study 2015. Popul Health Metrics.

[20] Marinho F, Passos VM, França EB (2016). New century, new challenges: changes in the burden of disease profile in Brazil, 1990-2010. Epidemiol Serv Saude.

[21] Ross JM, Henry NJ, Dwyer-Lindgren LA, de Paula LA, de Souza FM, Biehl MH (2018). Progress toward eliminating TB and HIV deaths in Brazil, 2001–2015: a spatial assessment. BMC Med.

[22] Ministério da Saúde. Secretaria de Vigilância em Saúde. Dados Epidemiológicos da Tuberculose no Brasil. http://portalarquivos2.saude.gov.br/images/pdf/2019/dezembro/09/APRES-PADRAO-NOV-19.pdf. Brasília: Ministério da Saúde; 2019. Accessed 20 Dec 2019.

[23] Nhamoyebonde S, Leslie A (2014). Biological differences between the sexes and susceptibility to tuberculosis. J Infect Dis.

[24] Moreira CM, Zandonade E, Reynaldo D, Maciel EL (2008). Tuberculosis-related mortality in the state of Espírito Santo, Brazil, 1985-2004. J Bras Pneumol.

[25] Horton KC, MacPherson P, Houben RM, White RG, Corbett EL (2016). Sex differences in tuberculosis burden and notifications in low-and middle-income countries: a systematic review and meta-analysis. PLoS Med.

[26] Oliwa JN, Karumbi JM, Marais BJ, Madhi SA, Graham SM (2015). Tuberculosis as a cause or comorbidity of childhood pneumonia in tuberculosis-endemic areas: a systematic review. Lancet Respir Med.

[27] Graham SM, Sismanidis C, Menzies HJ, Marais BJ, Detjen AK, Black RE (2014). Importance of tuberculosis control to address child survival. Lancet..

[28] Dodd PJ, Yuen CM, Sismanidis C, Seddon JA, Jenkins HE (2017). The global burden of tuberculosis mortality in children: a mathematical modelling study. Lancet Glob Health.

[29] Kendall EA (2017). Tuberculosis in children: under-counted and under-treated. Lancet Glob Health.

[30] Carvalho ACC, Cardoso CAA, Martire TM, Migliori GB, Sant’Anna CC (2018). Epidemiological aspects, clinical manifestations, and prevention of pediatric tuberculosis from the perspective of the End TB Strategy. J Bras Pneumol.

[31] Ministério da Saúde. Secretaria de Vigilância em Saúde. Indicadores prioritários para o monitoramento do Plano Nacional pelo Fim da Tuberculose como Problema de Saúde Pública no Brasil. Boletim Epidemiológico 2017;48(8):1–11. http://portalarquivos2.saude.gov.br/images/pdf/2017/marco/23/2017-V-48-N-8-Indicadores-priorit%2D%2Drios-para-o-monitoramento-do-Plano-Nacional-pelo-Fim-da-Tuberculose-como-Problema-de-Sa%2D%2Dde-P%2D%2Dblica-no-Brasil.pdf. Accessed 15 Dec 2018.

[32] Ceccon RF, Maffacciolli R, Burille A, Meneghel SN, Oliveira DLLC, Gerhardt TE (2017). Tuberculosis mortality in Brazilian capitals, 2008-2010. Epidemiol Serv Saude.

[33] Soares MLM, Amaral NACD, Zacarias ACP, Ribeiro LKNP (2017). Sociodemographic, clinical and epidemiological aspects of Tuberculosis treatment abandonment in Pernambuco, Brazil, 2001-2014. Epidemiol Serv Saude.

[34] Castro DB, Pinto RC, Albuquerque BC, Sadahiro M, Braga JU (2016). The socioeconomic factors and the indigenous component of tuberculosis in Amazonas. PLoS One.

[35] Barreira D (2018). The challenges to eliminating tuberculosis in Brazil. Epidemiol Serv Saude.

[36] Maciel ELN, Sales CMM, Bertolde AI, Reis-Santos B (2018). Can Brazil achieve the new World Health Organization global targets for tuberculosis control?. Epidemiol Serv Saude.

[37] Kritski A, Andrade KB, Galliez RM, Maciel ELN, Cordeiro-Santos M, Miranda SS (2018). Tuberculosis: renewed challenge in Brazil. Rev Soc Bras Med Trop.

[38] Ministério da Saúde. Secretaria de Vigilância em Saúde. Departamento de Vigilância das Doenças Transmissíveis. Brasil Livre da Tuberculose: Plano Nacional pelo Fim da Tuberculose como Problema de Saúde Pública. http://bvsms.saude.gov.br/bvs/publicacoes/brasil_livre_tuberculose_plano_nacional.pdf. Brasília: Ministério da Saúde; 2017. Accessed 15 Dec 2019.

[39] Oliosi JGN, Reis-Santos B, Locatelli RL, Sales CMM, da Silva Filho WG, da Silva KC (2019). Effect of the Bolsa Familia Programme on the outcome of tuberculosis treatment: a prospective cohort study. Lancet Glob Health.

[40] Torrens AW, Rasella D, Boccia D, Maciel EL, Nery JS, Olson ZD (2016). Effectiveness of a conditional cash transfer programme on TB cure rate: a retrospective cohort study in Brazil. Trans R Soc Trop Med Hyg.

[41] Barbosa IR, Costa ICC (2014). Epidemiological study of tuberculosis-HIV co-infection in northeastern Brazil. Rev Patol Trop.

[42] Lima MD, Martins-Melo FR, Heukelbach J, Alencar CH, Boigny RN, Ramos Júnior AN (2016). Mortality related to tuberculosis-HIV/AIDS co-infection in Brazil, 2000-2011: epidemiological patterns and time trends. Cad Saude Publica.

[43] Melo MC, Donalisio MR, Cordeiro RC (2017). Survival of patients with AIDS and co-infection with the tuberculosis bacillus in the South and Southeast regions of Brazil. Cien Saude Colet.

[44] Saraceni V, King BS, Cavalcante SC, Golub JE, Lauria LM, Moulton LH (2008). Tuberculosis as primary cause of death among AIDS cases in Rio de Janeiro. Brazil Int J Tuberc Lung Dis.

[45] Ministério da Saúde (2017). Secretaria de Vigilância em Saúde. Coinfecção TB-HIV no Brasil: panorama epidemiológico e atividades colaborativas. Boletim Epidemiológico.

[46] Vendramini SH, Santos NS, Santos Mde L, Chiaravalloti-Neto F, Ponce MA, Gazetta CE (2010). Spatial analysis of tuberculosis/HIV coinfection: its relation with socioeconomic levels in a city in south-eastern Brazil. Rev Soc Bras Med Trop.

[47] Torrens A, Bartholomay P, Silva S, Khogali M, Verdonck K, Bissell K (2016). HIV testing, antiretroviral therapy, and treatment outcomes in new cases of tuberculosis in Brazil, 2011. Rev Panam Salud Publica.

[48] Silva JB, Cardoso GC, Netto AR, Kritski AL (2015). The meanings of comorbidity for patients living with TB/HIV: implications in the treatment. Physis..

[49] Saraceni V, Benzaken AS, Pereira GFM, Andrade KB, Oliveira PB, Arakaki-Sanchez D (2018). Tuberculosis burden on AIDS in Brazil: A study using linked databases. PLoS One.

[50] Al-Rifai RH, Pearson F, Critchley JA, Abu-Raddad LJ (2017). Association between diabetes mellitus and active tuberculosis: A systematic review and meta-analysis. PLoS One.

[51] Rehm J, Samokhvalov AV, Neuman MG, Room R, Parry C, Lönnroth K (2009). The association between alcohol use, alcohol use disorders and tuberculosis (TB). A systematic review. BMC Public Health.

[52] O’Leary SM, Coleman MM, Chew WM, Morrow C, McLaughlin AM, Gleeson LE (2014). Cigarette smoking impairs human pulmonary immunity to Mycobacterium tuberculosis. Am J Respir Crit Care Med.

[53] Imtiaz S, Shield KD, Roerecke M, Samokhvalov AV, Lönnroth K, Rehm J (2017). Alcohol consumption as a risk factor for tuberculosis: meta-analyses and burden of disease. Eur Respir J.

[54] Melsew Y, Doan T, Gambhir M, Cheng A, McBryde E, Trauer J (2018). Risk factors for infectiousness of patients with tuberculosis: a systematic review and meta-analysis. Epidemiol Infect.

[55] Martins-Melo FR, Carneiro M, Ramos AN, Heukelbach J, Ribeiro ALP, Werneck GL (2018). The burden of Neglected Tropical Diseases in Brazil, 1990-2016: A subnational analysis from the Global Burden of Disease Study 2016. PLoS Negl Trop Dis.

[56] de Oliveira GP, Pinheiro RS, Coeli CM, Barreira D, Codenotti SB (2012). Mortality information system for identifying underreported cases of tuberculosis in Brazil. Rev Bras Epidemiol.

[57] Rocha MS, Oliveira GP, Aguiar FP, Saraceni V, Pinheiro RS (2015). What are the causes of death of patients with tuberculosis: multiple causes of death in a cohort of cases and a research proposal of presumed causes. Cad Saude Publica.

[58] Santos ML, Coeli CM, Batista JDL, Braga MC, Albuquerque MFPM (2018). Factors associated with underreporting of tuberculosis based on data from Sinan Aids and Sinan TB. Rev Bras Epidemiol.

