# Supplementary material for: The burden of tuberculosis and attributable risk factors in Brazil, 1990–2017: results from the Global Burden of Disease Study 2017
Source: Popul Health Metr. 2020 Sep 30;18(Suppl 1):10. doi: 10.1186/s12963-020-00203-6 (PMC7526097; doi:10.1186/s12963-020-00203-6)
Supplement: Supplementary file 1 — Additional file 1: Table S1. Number of DALYs and age-standardized DALY rates (per 100,000 inhabitants) from tuberculosis among HIV-negative individuals in Brazil and states in 1990 and 2017, with absolute percentage change between 1990 and 2017. Table S2. Number of YLLs and age-standardized YLL rates (per 100,000 inhabitants) from tuberculosis among HIV-negative individuals in Brazil and states in 1990 and 2017, with absolute percentage change between 1990 and 2017. Table S3. Number of YLDs and age-standardized YLD rates (per 100,000 inhabitants) from tuberculosis among HIV-negative individuals in Brazil and states in 1990 and 2017, with absolute percentage change between 1990 and 2017. DALYs = disability-adjusted life-years. YLLs = years of life lost. YLDs = years lived with disability. 95% UI = 95% uncertainty interval. [file 12963_2020_203_MOESM1_ESM.doc]

**Table S1.** Number of DALYs and age-standardized DALY rates (per 100,000 inhabitants) from tuberculosis among HIV-negative individuals in Brazil and states in 1990 and 2017, with absolute percentage change between 1990 and 2017.

| **Region/State** | **Number of DALYs (95% UI)** | | | **Age-standardized DALY rates (per 100,000) (95% UI)** | | |
| --- | --- | --- | --- | --- | --- | --- |
|  | **1990** | **2017** | **% Change 1990–2017** | **1990** | **2017** | **% Change 1990–2017** |
| **Brazil** | 398,447.4 (380,679.9-419,115.8) | 196,366.4 (189,645.2-202,394.5) | -50.7 | 300.55 (289.03-313.84) | 85.30 (82.30-88.03) | -71.6 |
| ***North*** |  |  |  |  |  |  |
| Acre | 1,642.0 (1,465.8-1,834.2) | 1,082.2 (987.4-1,182.9) | -34.1 | 484.16 (446.10-525.10) | 138.69 (126.70-151.61) | -71.4 |
| Amapá | 451.4 (409.9-494.8) | 714.6 (654.8-775.1) | 58.3 | 227.58 (211.73-246.53) | 99.91 (91.83-107.97) | -56.1 |
| Amazonas | 7,247.9 (6,538.8-8,087.1) | 5,722.6 (5,319.1-6,186.1) | -21.0 | 452.67 (420.10-487.39) | 159.84 (149.05-172.11) | -64.7 |
| Rondônia | 2,799.7 (2,478.0-3,152.4) | 1,429.1 (1,262.4-1,626.0) | -49.0 | 341.68 (309.03-378.14) | 81.48 (71.72-92.45) | -76.2 |
| Roraima | 515.6 (447.9-585.3) | 496.3 (430.3-569.1) | -3.7 | 320.42 (286.84-358.05) | 98.61 (85.95-112.30) | -69.2 |
| Pará | 15,756.8 (13,834.3-17,795.6) | 9,676.3 (8,943.9-10,474.2) | -38.6 | 383.86 (348.89-418.80) | 118.77 (109.91-128.54) | -69.1 |
| Tocantins | 1,392.0 (978.2-1,772.4) | 760.1 (678.2-844.3) | -45.4 | 172.33 (130.24-210.38) | 47.83 (42.63-53.16) | -72.2 |
| ***Northeast*** |  |  |  |  |  |  |
| Alagoas | 10,448.3 (8,525.5-12,725.6) | 3,647.2 (3,378.6-3,932.9) | -65.1 | 420.48 (358.91-488.23) | 104.44 (96.53-112.73) | -75.2 |
| Bahia | 45,684.7 (40,614.9-51,566.3) | 19,433.0 (18,138.6-20,830.2) | -57.5 | 429.16 (392.23-472.88) | 119.66 (111.43-128.75) | -72.1 |
| Ceará | 22,088.6 (18,180.8-26,785.5) | 10,153.3 (9,479.6-10,888.0) | -54.0 | 353.91 (301.94-411.38) | 101.23 (94.38-108.84) | -71.4 |
| Maranhão | 23,471.7 (19,699.7-28,568.5) | 8,064.7 (7,411.0-8,792.1) | -65.6 | 521.00 (443.14-625.00) | 111.16 (102.69-120.51) | -78.7 |
| Paraíba | 8,074.7 (6,750.8-9,541.8) | 3,466.1 (3,119.5-3,825.0) | -57.1 | 274.00 (235.30-317.54) | 77.34 (69.42-85.38) | -71.8 |
| Pernambuco | 34,884.2 (30,975.8-39,230.9) | 17,242.4 (16,222.9-18,389.3) | -50.6 | 528.43 (484.01-582.04) | 165.23 (155.59-176.40) | -68.7 |
| Piauí | 6,206.4 (5,037.8-7,484.9) | 3,107.2 (2,848.4-3,362.8) | -49.9 | 270.72 (227.17-321.13) | 85.05 (77.84-92.04) | -68.6 |
| Rio Grande do Norte | 6,184.2 (5,201.4-7,202.1) | 3,510.3 (3,232.5-3,803.2) | -43.2 | 263.42 (230.66-299.23) | 91.97 (84.40-99.71) | -65.1 |
| Sergipe | 3,520.0 (3,071.7-4,013.6) | 2,049.9 (1,880.4-2,224.8) | -41.8 | 261.79 (234.81-289.85) | 84.95 (77.96-92.17) | -67.5 |
| ***Southeast*** |  |  |  |  |  |  |
| Espírito Santo | 4,612.3 (4209.0-5,030.8) | 2,638.2 (2,434.2-2,834.7) | -42.8 | 208.50 (193.40-223.92) | 60.73 (56.03-65.47) | -70.9 |
| Minas Gerais | 23,993.7 (21,588.5-26,610.5) | 11,697.9 (10,939.9-12,513.8) | -51.2 | 172.88 (158.97-188.14) | 47.95 (44.58-51.63) | -72.3 |
| Rio de Janeiro | 60,347.1 (57,240.2-63,903.3) | 30,846.3 (28,993.5-32,663.7) | -48.9 | 479.00 (454.52-507.29) | 151.40 (141.96-160.51) | -68.4 |
| São Paulo | 74,251.3 (68,148.0-80,928.8) | 34,446.7 (32,188.5-36,709.6) | -53.6 | 252.09 (232.11-273.25) | 66.20 (61.76-70.73) | -73.7 |
| ***South*** |  |  |  |  |  |  |
| Paraná | 11,762.5 (10,573.3-13,109.6) | 5,781.4 (5,382.1-6,212.8) | -50.8 | 163.87 (150.13-179.21) | 45.34 (42.05-49.08) | -72.3 |
| Rio Grande do Sul | 16,554.0 (15,429.4-17,925.3) | 9,375.9 (8,743.2-10,070.3) | -43.4 | 194.08 (181.42-209.46) | 70.18 (65.50-75.40) | -63.8 |
| Santa Catarina | 3,916.9 (3,300.4-4,630.0) | 2,269.4 (2,088.3-2,454.2) | -42.1 | 102.72 (88.99-118.64) | 28.57 (26.08-31.51) | -72.2 |
| ***Central-West*** |  |  |  |  |  |  |
| Distrito Federal | 1,312.1 (1,133.5-1,503.1) | 760.2 (679.2-843.3) | -42.1 | 103.09 (92.66-113.94) | 24.68 (22.09-27.52) | -76.1 |
| Goiás | 3,840.4 (3,252.5-4,544.7) | 2,685.6 (2,462.0-2,911.8) | -42.8 | 113.19 (99.77-129.53) | 36.41 (33.18-39.71) | -67.8 |
| Mato Grosso | 4,475.9 (4,044.2-4,966.8) | 3,075.6 (2,830.9-3,330.7) | -31.3 | 282.78 (258.44-308.54) | 84.99 (78.11-92.23) | -69.9 |
| Mato Grosso do Sul | 3,012.9 (2,642.5-3,341.9) | 2,234.0 (2,059.9-2,426.5) | -25.9 | 207.68 (187.09-226.69) | 75.06 (69.09-81.28) | -63.9 |

DALYs = disability-adjusted life-years. 95% UI = 95% uncertainty interval.

**Table S2:** Number of YLLs and age-standardized YLL rates (per 100,000 inhabitants) from tuberculosis among HIV-negative individuals in Brazil and states in 1990 and 2017, with absolute percentage change between 1990 and 2017.

| **Region/State** | **Number of YLLs (95% UI)** | | | **Age-standardized YLL rates (per 100,000) (95% UI)** | | |
| --- | --- | --- | --- | --- | --- | --- |
|  | **1990** | **2017** | **% Change 1990–2017** | **1990** | **2017** | **% Change 1990–2017** |
| **Brazil** | **391,021.6 (373,838.2-411,515.7)** | **187,975.1 (182,091.3-193,287.7** | **-51.9** | **294.97 (283.70-307.98)** | **81.62 (78.98-83.98)** | **-72.3** |
| ***North*** |  |  |  |  |  |  |
| Acre | 1,614.3 (1,439.2-1,804.7) | 1,033.8 (936.5-1,131.9) | -36.0 | 475.69 (437.99-516.58) | 132.98 (120.49-144.87) | -72.0 |
| Amapá | 436.8 (396.5-480.4) | 676.8 (621.2-735.4) | 54.9 | 220.72 (205.12-239.36) | 95.04 (87.20-103.11) | -56.9 |
| Amazonas | 7,103.2 (6,396.9-7,939.3) | 5,436.1 (5,055.4-5,864.6) | -23.5 | 443.75 (411.57-478.55) | 152.42 (142.24-164.37) | -65.7 |
| Rondônia | 2,752.4 (2,435.2-3,097.8) | 1,375.8 (1,205.8-1,574.4) | -50.0 | 336.41 (303.84-372.66) | 78.54 (68.77-89.44) | -76.7 |
| Roraima | 504.9 (437.3-575.2) | 474.5 (410.1-545.7) | -6.0 | 314.22 (280.43-351.03) | 94.55 (81.98-107.95) | -69.9 |
| Pará | 15,488.7 (13,595.4-17,553.2) | 9,279.7 (8,567.1-10,082.4) | -40.1 | 377.04 (342.23-412.76) | 114.21 (105.65-124.08) | -69.7 |
| Tocantins | 1,363.2 (953.8-1,748.3) | 716.8 (636.5-799.9) | -47.4 | 168.39 (125.75-206.43) | 45.15 (40.04-50.49) | -73.2 |
| ***Northeast*** |  |  |  |  |  |  |
| Alagoas | 10,341.0 (8,413.6-12,633.6) | 3,527.2 (3,257.8-3,794.0) | -65.9 | 415.46 (353.48-483.45) | 101.07 (93.35-108.82) | -75.7 |
| Bahia | 45,086.1 (40,005.6-50,955.8) | 18,783.7 (17,477.4-20,209.4) | -58.3 | 423.22 (385.81-465.65) | 115.68 (107.54-124.91) | -72.7 |
| Ceará | 21,787.5 (17,891.3-26,459.5) | 9,726.5 (9,080.2-10,465.4) | -55.4 | 348.46 (297.09-404.57) | 97.03 (90.44-104.56) | -72.2 |
| Maranhão | 23,216.5 (19,383.5-28,343.9) | 7,765.4 (7,121.3-8,464.3) | -66.6 | 514.58 (435.12-620.50) | 107.19 (98.89-116.39) | -79.2 |
| Paraíba | 7,943.6 (6,616.3-9,398.4) | 3,322.6 (2,990.3-3,684.1) | -58.2 | 269.32 (230.77-312.92) | 74.12 (66.55-81.91) | -72.5 |
| Pernambuco | 34,470.9 (30,572.6-38,825.4) | 16,746.9 (15,744.7-17,901.7) | -51.4 | 521.87 (476.56-573.80) | 160.45 (150.84-171.49) | -69.3 |
| Piauí | 6,101.2 (4,940.7-7,381.5) | 2,974.8 (2,724.8-3,231.8) | -51.2 | 265.87 (222.97-315.49) | 81.44 (74.64-88.50) | -69.4 |
| Rio Grande do Norte | 6,088.9 (5,120.8-7,096.3) | 3,370.3 (3,089.4-3,665.2) | -44.6 | 258.95 (226.30-293.82) | 88.32 (80.84-96.09) | -65.9 |
| Sergipe | 3,462.3 (3,009.9-3,958.2) | 1,971.9 (1,807.6-2,138.2) | -43.0 | 257.24 (230.51-285.50) | 81.76 (74.97-88.75) | -68.2 |
| ***Southeast*** |  |  |  |  |  |  |
| Espírito Santo | 4,503.7 (4,114.5-4,918.9) | 2,517.4 (2,318.8-2,706.5) | -44.1 | 203.82 (188.77-219.27) | 57.89 (53.32-62.60) | -71.6 |
| Minas Gerais | 23,307.7 (20,844.3-25,837.8) | 11,003.4 (10,225.8-11,788.5) | -52.8 | 168.12 (154.05-183.25) | 45.00 (41.68-48.61) | -73.2 |
| Rio de Janeiro | 59,536.8 (56,417.7-63,094.9) | 29,842.8 (27,950.3-31,619.9) | -49.9 | 472.67 (448.15-500.20) | 146.29 (136.93-155.18) | -69.1 |
| São Paulo | 72,512.1 (66,148.3-79,115.1) | 32,750.8 (30,641.6-34,943.4) | -54.8 | 246.35 (226.14-267.70) | 62.81 (58.52-67.12) | -74.5 |
| ***South*** |  |  |  |  |  |  |
| Paraná | 11,398.1 (10,202.3-12,720.7) | 5,470.5 (5,091.1-5,901.0) | -52.0 | 159.13 (145.14-174.39) | 42.82 (39.74-46.58) | -73.1 |
| Rio Grande do Sul | 15,988.2 (14,877.4-17,283.1) | 8,871.5 (8,275.9-9,502.3) | -44.5 | 187.72 (174.74-202.51) | 66.21 (61.46-70.95) | -64.7 |
| Santa Catarina | 3,748.0 (3,137.2-4,426.4) | 2,078.8 (1,907.5-2,248.0) | -44.5 | 98.64 (84.75-114.34) | 26.09 (23.81-28.97) | -73.6 |
| ***Central-West*** |  |  |  |  |  |  |
| Distrito Federal | 1,256.4 (1,081.4-1,442.0) | 681.1 (606.2-759.8) | -45.8 | 99.13 (88.73-109.96) | 22.16 (19.82-25.00) | -77.7 |
| Goiás | 3,715.1 (3,128.7-4,419.6) | 2,527.0 (2,318.4-2,740.9) | -32.0 | 109.70 (96.05-126.19) | 34.23 (31.25-37.57) | -68.8 |
| Mato Grosso | 4,371.4 (3935.3-4,843.3) | 2,924.4 (2,680.5-3,171.0) | -33.1 | 276.49 (252.05-302.05) | 80.87 (73.93-88.05) | -70.8 |
| Mato Grosso do Sul | 2,922.6 (2,550.4-3,250.1) | 2,124.4 (1,950.9-2,311.8) | -27.3 | 201.88 (180.30-220.48) | 71.35 (65.38-77.42) | -64.7 |

YLLs = years of life lost. 95% UI = 95% uncertainty interval.

**Table S3.** Number of YLDs and age-standardized YLD rates (per 100,000 inhabitants) from tuberculosis among HIV-negative individuals in Brazil and states in 1990 and 2017, with absolute percentage change between 1990 and 2017.

| **Region/State** | **Number of YLDs (95% UI)** | | | **Age-standardized YLD rates (per 100,000) (95% UI)** | | |
| --- | --- | --- | --- | --- | --- | --- |
|  | **1990** | **2017** | **% Change 1990–2017** | **1990** | **2017** | **% Change 1990–2017** |
| **Brazil** | **7,425.8 (4,943.8-10,153.7)** | **8,391.3 (5,533.4-11,692.8)** | **13.0** | **5.58 (3.72-7.57)** | **3.68 (2.43-5.12)** | **-34.1** |
| ***North*** |  |  |  |  |  |  |
| Acre | 27.7 (17.9 -39.3) | 48.3 (30.8-70.2) | 74.6 | 8.47 (5.64-11.79) | 5.71 (3.67-8.16) | -32.6 |
| Amapá | 14.5 (9.4-21.2) | 37.9 (24.3-53.8) | 160.4 | 6.85 (4.56-9.68) | 4.86 (3.20-6.80) | -29.0 |
| Amazonas | 144.7 (91.8-205.4) | 286.5 (183.4-404.0) | 98.0 | 8.93 (5.87-12.51) | 7.42( 4.84-10.34) | -16.9 |
| Rondônia | 47.3 (29.5-68.5) | 53.2 (33.0-78.6) | 12.5 | 5.27 (3.35-7.41) | 2.94 (1.84-4.28) | -44.1 |
| Roraima | 10.6 (6.9-15.4) | 21.8 (13.4-31.4) | 105.4 | 6.20 (4.06-8.88) | 4.06 (2.55-5.73) | -34.6 |
| Pará | 268.1 (170.3-388.1) | 396.6 (251.2-577.8) | 47.9 | 6.82 (4.45-9.73) | 4.56 (2.91-6.63) | -33.2 |
| Tocantins | 28.8 (18.5-41.8) | 43.2 (27.7-62.2) | 50.0 | 3.94 (2.52-5.59) | 2.67 (1.70-3.82) | -32.1 |
| ***Northeast*** |  |  |  |  |  |  |
| Alagoas | 107.3 (69.9-154.1) | 120.0 (75.3-176.1) | 11.8 | 5.03 (3.32-7.22) | 3.37 (2.13-4.94) | -32.9 |
| Bahia | 598.6 (387.0-853.4) | 649.3 (408.9-941.0) | 8.5 | 5.94 (3.90-8.30) | 3.98 (2.50-5.76) | -33.1 |
| Ceará | 301.1 (198.5-433.3) | 426.8 (273.8-604.3) | 41.7 | 5.45 (3.59-7.78) | 4.20 (2.72-5.93) | -22.9 |
| Maranhão | 255.2 (163.5-363.6) | 299.3 (184.5-433.1) | 17.3 | 6.42 (4.14-9.14) | 3.97 (2.50-5.70) | -38.3 |
| Paraíba | 131.2 (85.0-189.0) | 143.5 (91.0-209.4) | 9.4 | 4.68 (3.01-6.73) | 3.22 (2.04-4.68) | -31.1 |
| Pernambuco | 413.3 (270.9-578.1) | 495.5 (316.4-714.9)( | 19.9 | 6.56 (4.36-9.12) | 4.78 (3.06-6.91) | -27.2 |
| Piauí | 105.2 (68.1-149.4) | 132.4 (82.9-189.5) | 25.9 | 4.85 (3.13-6.88) | 3.62 (2.26-5.16) | -25.4 |
| Rio Grande do Norte | 95.2 (61.8-135.4) | 140.0 (88.3-206.7) | 47.0 | 4.47 (2.92-6.39) | 3.65 (2.31-5.36) | -18.2 |
| Sergipe | 57.7 (37.4-82.2) | 78.1 (49.4-110.8) | 35.3 | 4.55 (2.94-6.41) | 3.19 (2.02-4.52) | -30.0 |
| ***Southeast*** |  |  |  |  |  |  |
| Espírito Santo | 108.6 (69.2-157.7) | 120.7 (76.4-170.3) | 11.2 | 4.68 (3.06-6.72) | 2.84 (1.81-4.01) | -39.4 |
| Minas Gerais | 686.0 (433.9-988.4) | 694.5 (441.9-1,001.7) | 1.2 | 4.76 (3.05-6.72) | 2.94 (1.87-4.23) | -38.1 |
| Rio de Janeiro | 810.4 (527.8-1,147.1) | 1,003.5 (646.7-1,447.0) | 23.8 | 6.33 (4.17-8.92) | 5.12 (3.31-7.33) | -19.2 |
| São Paulo | 1,739.2 (1,086.3-2,432.8) | 1,695.9 (1,092.5-2,447.9) | -2.5 | 5.74 (3.69-7.96) | 3.39 (2.19-4.89) | -40.9 |
| ***South*** |  |  |  |  |  |  |
| Paraná | 364.4 (230.7-530.4) | 310.9 (192.9-441.1) | -14.7 | 4.75 (3.07-6.85) | 2.52 (1.58-3.55) | -47.0 |
| Rio Grande do Sul | 565.9 (365.3-808.9) | 504.4 (323.6-727.0) | -10.9 | 6.36 (4.23-9.10) | 3.96 (2.53-5.78) | -37.7 |
| Santa Catarina | 169.0 (106.4-244.0) | 190.6 (120.8-277.2) | 12.8 | 4.08 (2.61-5.83) | 2.48 (1.57-3.62) | -39.3 |
| ***Central-West*** |  |  |  |  |  |  |
| Distrito Federal | 55.7 (36.2-80.9) | 79.1 (49.4-115.6) | 42.1 | 3.96 (2.66-5.66) | 2.53 (1.59-3.65) | -36.2 |
| Goiás | 125.3 (80.2-181.2) | 158.5 (99.5-230.8) | 26.5 | 3.49 (2.28-5.01) | 2.18 (1.38-3.15) | -37.7 |
| Mato Grosso | 104.5 (67.1-149.0) | 151.1 (94.0-221.5) | 44.6 | 5.81 (3.74-8.07) | 4.12 (2.60-6.00) | -34.6 |
| Mato Grosso do Sul | 90.3 (55.9-128.2) | 109.6 (69.6-158.0) | 21.4 | 6.29 (4.04-8.84) | 3.71 (2.38-5.33) | -36.1 |

YLDs = years lived with disability. 95% UI = 95% uncertainty interval.
