# Supplementary material for: The burden of tuberculosis and attributable risk factors in Brazil, 1990–2017: results from the Global Burden of Disease Study 2017
Source: Popul Health Metr. 2020 Sep 30;18(Suppl 1):10. doi: 10.1186/s12963-020-00203-6 (PMC7526097; doi:10.1186/s12963-020-00203-6)
Supplement: Supplementary file 2 — Additional file 2: Table S1. Number of DALYs and age-standardized DALY rates (per 100,000 inhabitants) from tuberculosis among HIV-positive individuals in Brazil and states in 1990 and 2017, with absolute percentage change between 1990 and 2017. Table S2. Number of YLLs and age-standardized YLL rates (per 100,000 inhabitants) from tuberculosis among HIV-positive individuals in Brazil and states in 1990 and 2017, with absolute percentage change between 1990 and 2017. Table S3. Number of YLDs and age-standardized YLD rates (per 100,000 inhabitants) from tuberculosis among HIV-positive individuals in Brazil and states in 1990 and 2017, with absolute percentage change between 1990 and 2017. DALYs = disability-adjusted life-years. YLLs = years of life lost. YLDs = years lived with disability. 95% UI = 95% uncertainty interval. [file 12963_2020_203_MOESM2_ESM.doc]

**Table S1**. Number of DALYs and age-standardized DALY rates (per 100.000 inhabitants) from tuberculosis among HIV-positive individuals in Brazil and states in 1990 and 2017, with absolute percentage change between 1990 and 2017.

| **Region/State** | **Number of DALYs (95% UI)** | | | **Age-standardized DALY rates (per 100.000) (95% UI)** | | |
| --- | --- | --- | --- | --- | --- | --- |
|  | **1990** | **2017** | **% Change 1990–2017** | **1990** | **2017** | **% Change 1990–2017** |
| **Brazil** | **137,534.5 (88508.9-185,136.8)** | **87,957.1 (50,623.8-146,870.3)** | **-36.0** | **91.13 (58.61-122.70)** | **38.23 (21.99-63.81)** | **-58.0** |
| ***North*** |  |  |  |  |  |  |
| Acre | 335.7 (256.6-373.8) | 352.4 (178.2-641.0) | 5.0 | 77.30 (59.14-85.62) | 39.60 (20.05-72.03) | -48.8 |
| Amapá | 113.7 (72.1-160.7) | 228.2 (110.5-410.7) | 100.8 | 44.66 (28.31-63.23) | 26.72 (12.94-48.07) | -40.2 |
| Amazonas | 1,618.2 (1,153.5-1,830.6) | 2,878.4 (1,443.3-5,335.3) | 77.9 | 78.68 (56.12-88.63) | 68.48 (34.36-126.94) | -13.0 |
| Rondônia | 677.8 (447.7-853.7) | 511.5 (250.7-931.1) | -24.5 | 60.23 (39.80-75.49) | 26.47 (12.97-48.20) | -56.0 |
| Roraima | 194.2 (119.4-298.3) | 200.3 (105.0-367.1) | 3.1 | 80.86 (49.74-124.03) | 33.86 (17.76-62.06) | -58.1 |
| Pará | 3,230.5 (2,077.2-4,081.20 | 4,153.3 (2,190.5-7,508.6) | 28.6 | 61.30 (39.44-77.19) | 44.87 (23.69-81.10) | -26.8 |
| Tocantins | 241.6 (149.2-372.6) | 253.3 (123.8-465.4) | 4.8 | 25.17 (15.57-38.77) | 15.03 (7.34-27.61) | -40.3 |
| ***Northeast*** |  |  |  |  |  |  |
| Alagoas | 2,025.3 (1,443.9-2,338.4) | 1,399.3 (695.8-2,634.6) | -30.9 | 70.65 (50.41-80.77) | 38.46 (19.13-72.43) | -45.6 |
| Bahia | 10,172.9 (6,593.9-12,620.5) | 7,033.2 (3,668.8-12,844.1) | -30.9 | 76.82 (49.85-94.89) | 42.81 (22.33-78.18) | -44.3 |
| Ceará | 4,830.2 (3,032.3-6,202.8) | 4,103.6 (2,016.4-7,536.9) | -15.0 | 70.47 (44.22-90.38) | 40.34 (19.82-74.11) | -42.8 |
| Maranhão | 2,779.4 (2,277.3-3,056.0) | 3,192.3 (1,659.6-5,812.4) | 14.9 | 51.74 (42.35-56.43) | 40.21 (20.92-73.18) | -22.3 |
| Paraíba | 1,023.8 (821.7-1,121.4) | 1,313.4 (671.3-2,381.6) | 28.3 | 33.20 (26.67-36.21) | 29.46 (15.05-53.42) | -11.3 |
| Pernambuco | 11,934.9 (7,747.2-14,829.2) | 7,635.0 (3,935.0-13,841.9) | -36.0 | 144.91 (94.17-179.22) | 72.10 (37.16-130.74) | -50.2 |
| Piauí | 1,251.7 (767.2-1,858.3) | 1,227.6 (640.7-2,262.8) | -1.9 | 43.23 (26.54-63.85) | 33.24 (17.35-61.28) | -23.1 |
| Rio Grande do Norte | 1,219.6 (761.3-1,683.0) | 1,144.8 (578.9-2,100.0) | -6.1 | 45.57 (28.45-62.94) | 29.81 (15.06-54.69) | -34.6 |
| Sergipe | 882.6 (557.3-1,162.4) | 715.5 (353.9-1,321.4) | -18.9 | 59.08 (37.31-77.84) | 28.69 (14.18-53.00) | -51.4 |
| ***Southeast*** |  |  |  |  |  |  |
| Espírito Santo | 1,319.0 (820.8-1,925.5) | 1,118.2 (573.4-2,028.5) | -15.2 | 49.37 (30.72-71.97) | 26.18 (13.41-47.51) | -47.0 |
| Minas Gerais | 6,736.4 (4,312.5-9,911.0) | 4,614.4 (2,510.7-8,369.7) | -31.5 | 41.85 (26.79-61.73) | 19.52 (10.61-35.43) | -53.4 |
| Rio de Janeiro | 31,054.1 (20,301.7-36,585.0) | 18,140.6 (9,313.9-33,642.3) | -41.6 | 227.27 (148.63-267.54) | 93.08 (47.76-172.66) | -59.0 |
| São Paulo | 42,025.0 (25,925.9-65,147.0) | 14,729.6 (7,823.7-26,298.5) | -65.0 | 121.76 (75.12-188.79) | 28.45 (15.09-50.81) | -76.6 |
| ***South*** |  |  |  |  |  |  |
| Paraná | 3,103.6 (1,923.4-4,670.5) | 2,382.2 (1,152.1-4,360.6) | -23.2 | 35.42 (21.94-53.32) | 19.00 (9.18-34.81) | -46.4 |
| Rio Grande do Sul | 5,685.8 (3,597.8-8,464.3) | 5,910.6 (2,935.7-10,771.1) | 4.0 | 59.42 (37.58-88.44) | 46.50 (23.08-84.79) | -21.7 |
| Santa Catarina | 1,305.9 (812.9-2,007.5) | 1,130.7 (589.3-2,113.7) | -13.4 | 28.04 (17.44-43.13) | 14.14 (7.36-26.47) | -49.6 |
| ***Central-West*** |  |  |  |  |  |  |
| Distrito Federal | 421.3 (257.8-649.8) | 268.0 (119.3-510.7) | -36.4 | 25.20 (15.40-38.87) | 8.05 (3.58-15.36) | -68.1 |
| Goiás | 1,124.0 (687.9-1,729.5) | 1,002.2 (535.5-1,805.8) | -10.8 | 27.43 (16.78-42.22) | 13.10 (6.99-23.61) | -52.2 |
| Mato Grosso | 1,238.4 (780.2-1,852.0) | 1,307.0 (653.6-2,462.3) | 5.5 | 59.55 (37.53-89.21) | 33.25 (16.61-62.65) | -44.2 |
| Mato Grosso do Sul | 988.9 (621.1-1,488.4) | 1,011.4 (530.2-1,847.3) | 2.3 | 54.80 (34.41-82.62) | 33.16 (17.37-60.60) | -39.5 |

DALYs = disability-adjusted life-years. 95% UI = 95% uncertainty interval.

**Table S2.** Number of YLLs and age-standardized YLL rates (per 100.000 inhabitants) from tuberculosis among HIV-positive individuals in Brazil and states in 1990 and 2017, with absolute percentage change between 1990 and 2017.

| **Region/State** | **Number of YLLs (95% UI)** | | | **Age-standardized YLL rates (per 100.000 population) (95% UI)** | | | |
| --- | --- | --- | --- | --- | --- | --- | --- |
|  | **1990** | **2017** | **% Change 1990–2017** | **1990** | **2017** | **% Change 1990–2017** | **ARC 1990-2017 (%)** |
| **Brazil** | **135,929.5 (87,191.1-183,569.5)** | **85,662.2 (48,349.6-144,845.8)** | **-37.0** | **90.07 (57.68-121.66)** | **37.26 (21.04-62.96)** | **-58.6** | **-4.8** |
| ***North*** |  |  |  |  |  |  |  |
| Acre | 331.7 (252.2-369.3) | 342.6 (168.7-629.4) | 3.3 | 76.23 (57.93-84.62) | 38.48 (18.95-70.69) | -49.5 | -4.7 |
| Amapá | 111.5 (70.2-158.9) | 219.5 (103.9-400.3) | 96.8 | 43.76 (27.53-62.41) | 25.67 (12.15-46.82) | -41.3 | -3.1 |
| Amazonas | 1,594.3 (1,126.4-1,807.7) | 2,788.3 (1,352.0-5,221.1) | 74.9 | 77.41 (54.67-87.44) | 66.30 (32.14-124.17) | -14.4 | -4.0 |
| Rondônia | 670.1 (439.7-846.2) | 499.6 (240.2-915.8) | -25.4 | 59.52 (39.06-74.81) | 25.86 (12.43-47.40) | -56.6 | -5.4 |
| Roraima | 191.8 (117.2-296.2) | 194.2 (99.5 -360.0) | 1.3 | 79.74 (48.72-122.99) | 32.80 (16.79-60.79) | -58.9 | -4.5 |
| Pará | 3,195.2 (2,048.1-4,041.4) | 4,029.9 (2,070.1-7,380.6) | 26.1 | 60.52 (38.80-76.26) | 43.50 (22.35-79.69) | -28.1 | -4.4 |
| Tocantins | 238.0 (145.0-369.0) | 244.3 (116.2-454.2) | 2.7 | 24.72 (15.06-38.33) | 14.49 (6.89-26.94) | -41.4 | -4.9 |
| ***Northeast*** |  |  |  |  |  |  |  |
| Alagoas | 2,010.9 (1,432.9-2,323.9) | 1,370.8 (663.6-2,594.5) | -31.8 | 70.02 (49.86-80.02) | 37.68 (18.24-71.32) | -46.2 | -5.2 |
| Bahia | 10,090.9 (6,499.6-12,540.9) | 6,883.8 (3,532.3-12,674.3) | -31.8 | 76.08 (48.99-94.02) | 41.92 (21.51-77.19) | -44.9 | -4.8 |
| Ceará | 4,785.1 (2,995.3-6,155.8) | 4,002.9 (1,930.0-7,401.3) | -16.3 | 69.71 (43.60-89.56) | 39.36 (18.98-72.79) | -43.5 | -4.7 |
| Maranhão | 2,747.5 (2,245.6-3,026.0) | 3,108.0 (1,554.1-5,725.5) | 13.1 | 51.01 (41.63-55.77) | 39.10 (19.55-72.02) | -23.4 | -5.8 |
| Paraíba | 1,009.8 (808.3-1,107.8) | 1,281.3 (648.3-2,343.5) | 26.9 | 32.72 (26.21-35.78) | 28.75 (14.55-52.58) | -12.1 | -4.8 |
| Pernambuco | 11,862.7 (7,663.7-14,757.9) | 7,494.0 (3,787.4-13,678.2) | -36.8 | 143.86 (92.94-178.16) | 70.78 (35.77-129.20) | -50.8 | -4.4 |
| Piauí | 1,238.2 (753.1-1,845.4) | 1,195.4 (614.9-2,221.1) | -3.5 | 42.65 (25.94-63.32) | 32.38 (16.65-60.16) | -24.1 | -4.4 |
| Rio Grande do Norte | 1,208.0 (753.1-1,672.9) | 1,117.4 (556.6-2,063.0) | -7.5 | 45.06 (28.08-62.48) | 29.11 (14.50-53.75) | -35.4 | -4.0 |
| Sergipe | 873.7 (548.1-1,153.1) | 698.2 (341.1-1,299.6) | -20.1 | 58.43 (36.65-77.08) | 28.01 (13.67-52.13) | -52.1 | -4.3 |
| ***Southeast*** |  |  |  |  |  |  |  |
| Espírito Santo | 1,301.5 (805.4-1,909.4) | 1,089.9 (545.2-1,992.0) | -16.3 | 48.72 (30.15-71.39) | 25.54 (12.77-46.68) | -47.6 | -4.7 |
| Minas Gerais | 6,627.1 (4,194.3-9,800.8) | 4,457.0 (2,372.9-8,190.6) | -32.7 | 41.18 (26.06-61.02) | 18.88 (10.05-34.70) | -54.2 | -4.9 |
| Rio de Janeiro | 30,787.9 (19,947.4-36,272.9) | 17,790.5 (9,073.3-33,325.7) | -42.2 | 225.40 (146.13-265.46) | 91.31 (46.53-171.06) | -59.5 | -4.3 |
| São Paulo | 41,444.4 (25,401.9-64,623.6) | 14,283.4 (7,428.2-25,875.9) | -65.5 | 120.12 (73.62-187.30) | 27.60 (14.35-50.00) | -77.0 | -5.1 |
| ***South*** |  |  |  |  |  |  |  |
| Paraná | 3,050.1 (1,865.8-4,618.0) | 2,307.5 (1,097.8-4,251.3) | -24.3 | 34.83 (21.31-52.64) | 18.42 (8.76-33.96) | -47.1 | -4.9 |
| Rio Grande do Sul | 5,578.1 (3,488.2-8,357.5) | 5,724.2 (2,735.9-10,596.5) | 2.6 | 58.35 (36.49-87.37) | 45.07 (21.54-83.43) | -22.8 | -3.9 |
| Santa Catarina | 1,274.9 (779.4-1,976.2) | 1,074.0 (541.1-2,046.5) | -15.8 | 27.42 (16.76-42.51) | 13.46 (6.78-25.65) | -50.9 | -4.9 |
| ***Central-West*** |  |  |  |  |  |  |  |
| Distrito Federal | 410.9 (246.7-637.9) | 251.8 (106.9-490.8) | -38.7 | 24.61 (14.77-38.20) | 7.58 (3.22-14.78) | -69.2 | -5.6 |
| Goiás | 1,103.2 (669.8-1,706.3) | 966.7 (505.9-1,751.5) | -12.4 | 26.94 (16.36-41.67) | 12.64 (6.62-22.91) | -53.1 | -4.3 |
| Mato Grosso | 1,219.9 (757.8-1,832.8) | 1,265.4 (616.8-2,416.4) | 3.7 | 58.64 (36.42-88.11) | 32.19 (15.69-61.48) | -45.1 | -4.6 |
| Mato Grosso do Sul | 971.9 (605.9-1,470.8) | 981.6 (496.7-1,810.8) | 1.0 | 53.88 (33.59-81.60) | 32.20 (16.30-59.40) | -40.2 | -3.9 |

YLLs = years of life lost. 95% UI = 95% uncertainty interval.

**Table S3.** Number of YLDs and age-standardized YLD rates (per 100.000 inhabitants) from tuberculosis among HIV-positive individuals in Brazil and states in 1990 and 2017, with absolute percentage change between 1990 and 2017.

| **Region/State** | **Number of YLDs (95% UI)** | | | **Age-standardized YLD rates (per 100.000) (95% UI)** | | |
| --- | --- | --- | --- | --- | --- | --- |
|  | **1990** | **2017** | **% Change 1990–2017** | **1990** | **2017** | **% Change 1990–2017** |
| **Brazil** | **1,605.1 (1,078.3-2,241.8)** | **2,294.8 (1,469.5-3,270.5)** | **43.0** | **1.06 (0.71-1.47)** | **0.97 (0.62-1.38)** | **-8.7** |
| ***North*** |  |  |  |  |  |  |
| Acre | 3.9 (2.7-5.4) | 9.8 (6.2-14.7) | 147.9 | 1.07 (0.73-1.47) | 1.12 (0.71-1.69) | 4.8 |
| Amapá | 2.2 (1.4-3.0) | 8.7 (5.5-13.2) | 305.1 | 0.89 (0.59-1.23) | 1.05 (0.66-1.58) | 17.7 |
| Amazonas | 23.9 (15.9-33.0) | 90.1 (54.2-147.8) | 277.5 | 1.27 (0.85-1.76) | 2.19 (1.33-3.54) | 71.8 |
| Rondônia | 7.6 (5.0-10.5) | 11.9 (7.5-17.9) | 55.6 | 0.70 (0.46-0.97) | 0.61 (0.39-0.93) | -12.9 |
| Roraima | 2.4 (1.6-3.3) | 6.1 (3.8-9.1) | 151.2 | 1.12 (0.74-1.54) | 1.06 (0.66-1.59) | -5.4 |
| Pará | 35.4 (23.6-49.0) | 123.4 (78.3-186.5) | 249.0 | 0.78 (0.52-1.08) | 1.37 (0.87-2.07) | 74.9 |
| Tocantins | 3.7 (2.4-5.1) | 9.0 (5.6-13.7) | 145.7 | 0.44 (0.29-0.61) | 0.54 (0.33-0.82) | 20.8 |
| ***Northeast*** |  |  |  |  |  |  |
| Alagoas | 14.4 (9.6-19.8) | 28.5 (17.5-43.3) | 97.9 | 0.63 (0.42-0.87) | 0.78 (0.48-1.20) | 24.1 |
| Bahia | 82.0 (54.3-113.0) | 149.3 (93.5-224.2) | 82.2 | 0.74 (0.49-1.02) | 0.89 (0.56-1.34) | 20.0 |
| Ceará | 45.2 (29.7-62.0) | 100.7 (64.2-154.6) | 123.1 | 0.76 (0.50-1.04) | 0.98 (0.63-1.51) | 29.0 |
| Maranhão | 31.9 (21.3-43.7) | 84.3 (53.4-126.6) | 164.2 | 0.73 (0.49-1.00) | 1.11 (0.70-1.67) | 51.4 |
| Paraíba | 14.0 (9.2-19.3) | 32.2 (20.4-47.9) | 129.3 | 0.48 (0.32-0.67) | 0.71 (0.45-1.06) | 47.0 |
| Pernambuco | 72.2 (45.6-109.4) | 141.0 (89.9-211.8) | 95.3 | 1.05 (0.66-1.59) | 1.32 (0.84-1.99) | 26.5 |
| Piauí | 13.5 (9.0-18.5) | 32.2 (20.1-48.4) | 138.7 | 0.58 (0.39-0.79) | 0.86 (0.54-1.30) | 50.0 |
| Rio Grande do Norte | 11.6 (7.6-16.1) | 27.5 (17.3-41.4) | 137.7 | 0.51 (0.34-0.71) | 0.70 (0.44-1.06) | 37.9 |
| Sergipe | 8.9 (5.9-12.3) | 17.3 (10.8-26.4) | 93.6 | 0.65 (0.43-0.89) | 0.68 (0.43-1.04) | 5.1 |
| ***Southeast*** |  |  |  |  |  |  |
| Espírito Santo | 17.4 (11.3-24.1) | 28.3 (17.8-42.9) | 62.6 | 0.65 (0.42-0.90) | 0.64 (0.40-0.97) | -1.6 |
| Minas Gerais | 109.3 (71.7-150.3) | 157.3 (99.3-237.3) | 44.0 | 0.67 (0.44-0.92) | 0.64 (0.40-0.96) | -4.5 |
| Rio de Janeiro | 266.2 (160.5-391.0) | 350.1 (217.6-544.1) | 31.5 | 1.87 (1.14-2.74) | 1.77 (1.08-2.77) | -5.7 |
| São Paulo | 580.6 (348.0-866.5) | 446.2 (282.0-660.6) | -23.1 | 1.64 (1.00-2.43) | 0.85 (0.54-1.25) | -48.4 |
| ***South*** |  |  |  |  |  |  |
| Paraná | 53.5 (35.0-73.7) | 74.7 (47.1-112.6) | 39.5 | 0.59 (0.39-0.81) | 0.58 (0.36-0.87) | -2.3 |
| Rio Grande do Sul | 107.7 (70.9-149.4) | 186.4 (118.2-281.3) | 73.0 | 1.08 (0.71-1.49) | 1.44 (0.91-2.17) | 33.4 |
| Santa Catarina | 30.9 (20.3-43.3) | 56.7 (34.4-86.8) | 83.4 | 0.62 (0.41-0.87) | 0.69 (0.42-1.05) | 10.9 |
| ***Central-West*** |  |  |  |  |  |  |
| Distrito Federal | 10.4 (6.9-14.5) | 16.2 (9.9-25.6) | 55.7 | 0.59 (0.39-0.82) | 0.47 (0.29-0.74) | -20.5 |
| Goiás | 20.9 (13.8-29.1) | 35.5 (22.3-53.7) | 70.2 | 0.49 (0.32-0.68) | 0.46 (0.29-0.69) | -7.0 |
| Mato Grosso | 18.5 (12.2-25.5) | 41.6 (25.9-63.6) | 124.5 | 0.91 (0.60-1.26) | 1.06 (0.66-1.61) | 15.7 |
| Mato Grosso do Sul | 17.0 (11.1-23.4) | 29.8 (18.8-45.4) | 75.7 | 0.92 (0.60-1.27) | 0.96 (0.61-1.46) | 4.3 |

YLDs = years lived with disability. 95% UI = 95% uncertainty interval.
