# Supplementary figures and images for: The burden of tuberculosis and attributable risk factors in Brazil, 1990–2017: results from the Global Burden of Disease Study 2017
Source: Popul Health Metr. 2020 Sep 30;18(Suppl 1):10. doi: 10.1186/s12963-020-00203-6 (PMC7526097; doi:10.1186/s12963-020-00203-6)

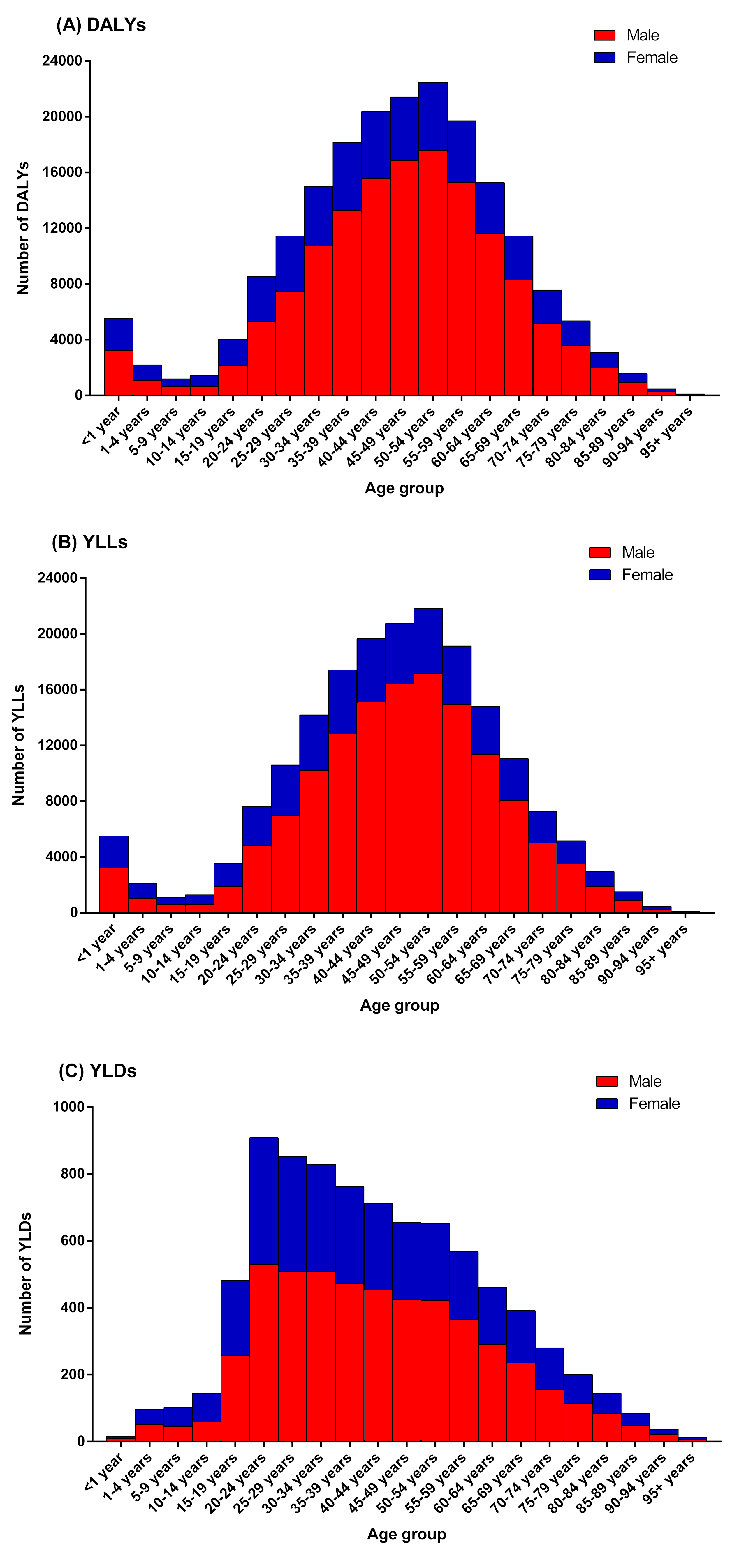

Supplement: Supplementary file 3 — Additional file 3: Figure S1. Absolute number of age- and sex-specific (A) DALYs, (B) YLLs, and (C) YLDs from tuberculosis among HIV-negative individuals in Brazil, 2017. DALYs = disability-adjusted life-years; YLLs = years of life lost; YLDs = years lived with disability. [file 12963_2020_203_MOESM3_ESM.tif]

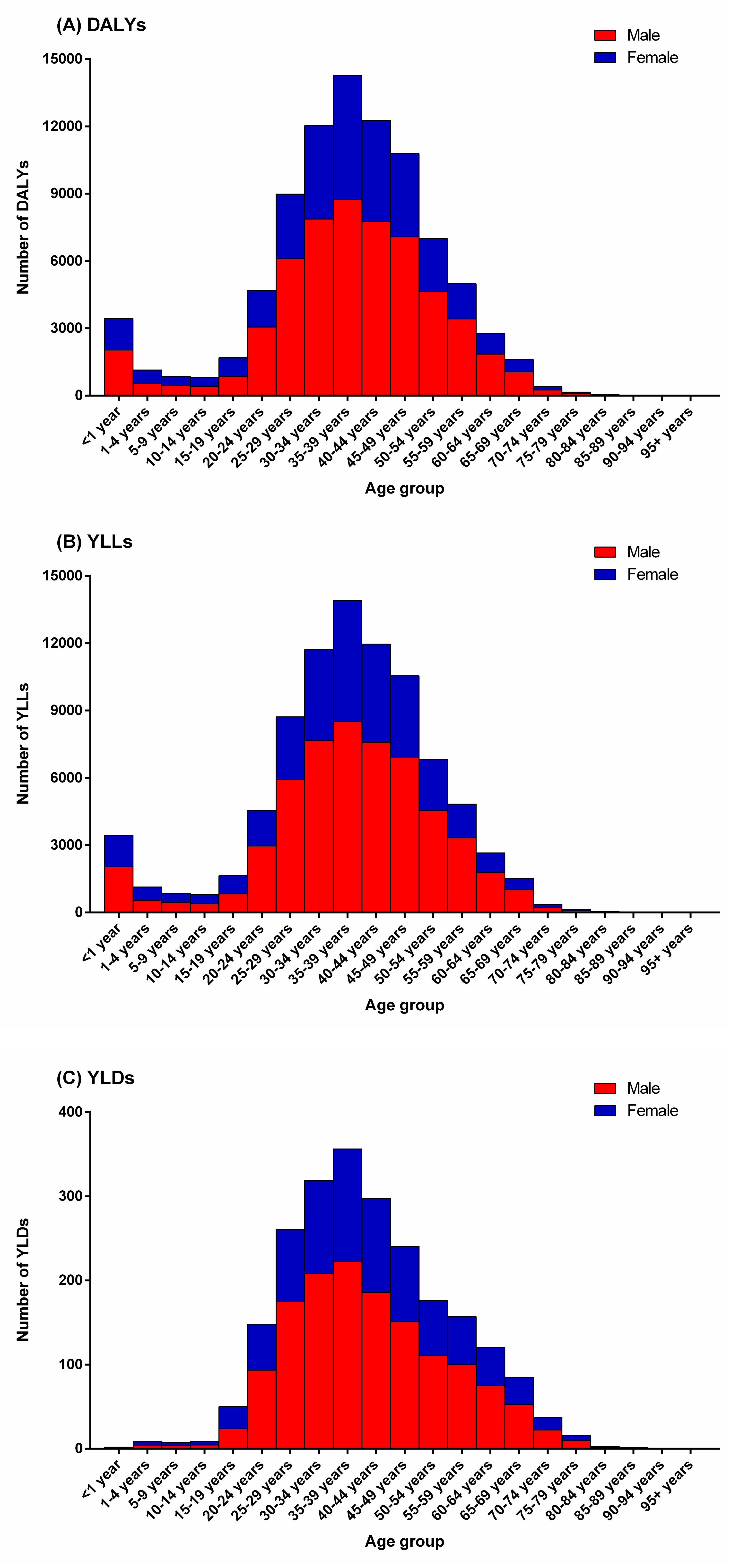

Supplement: Supplementary file 4 — Additional file 4: Figure S2. Absolute number of age- and sex-specific (A) DALYs, (B) YLLs, and (C) YLDs from tuberculosis among HIV-positive individuals in Brazil, 2017. DALYs = disability-adjusted life-years; YLLs = years of life lost; YLDs = years lived with disability. [file 12963_2020_203_MOESM4_ESM.tif]

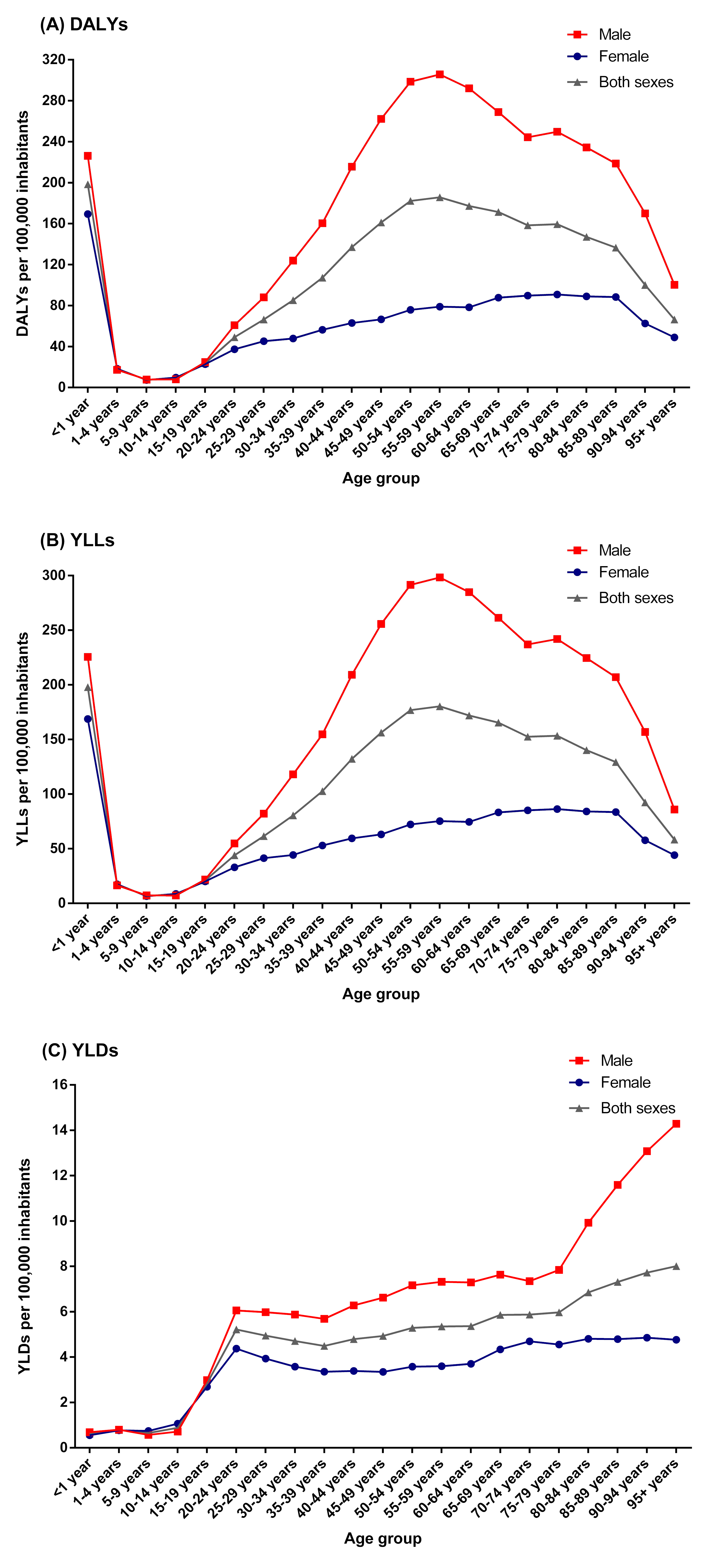

Supplement: Supplementary file 5 — Additional file 5: Figure S3. Age- and sex-specific rates (per 100,000 inhabitants) of (A) DALYs, (B) YLLs, and (C) YLDs from tuberculosis among HIV-negative individuals in Brazil, 2017. DALYs = disability-adjusted life-years; YLLs = years of life lost; YLDs = years lived with disability. [file 12963_2020_203_MOESM5_ESM.tif]

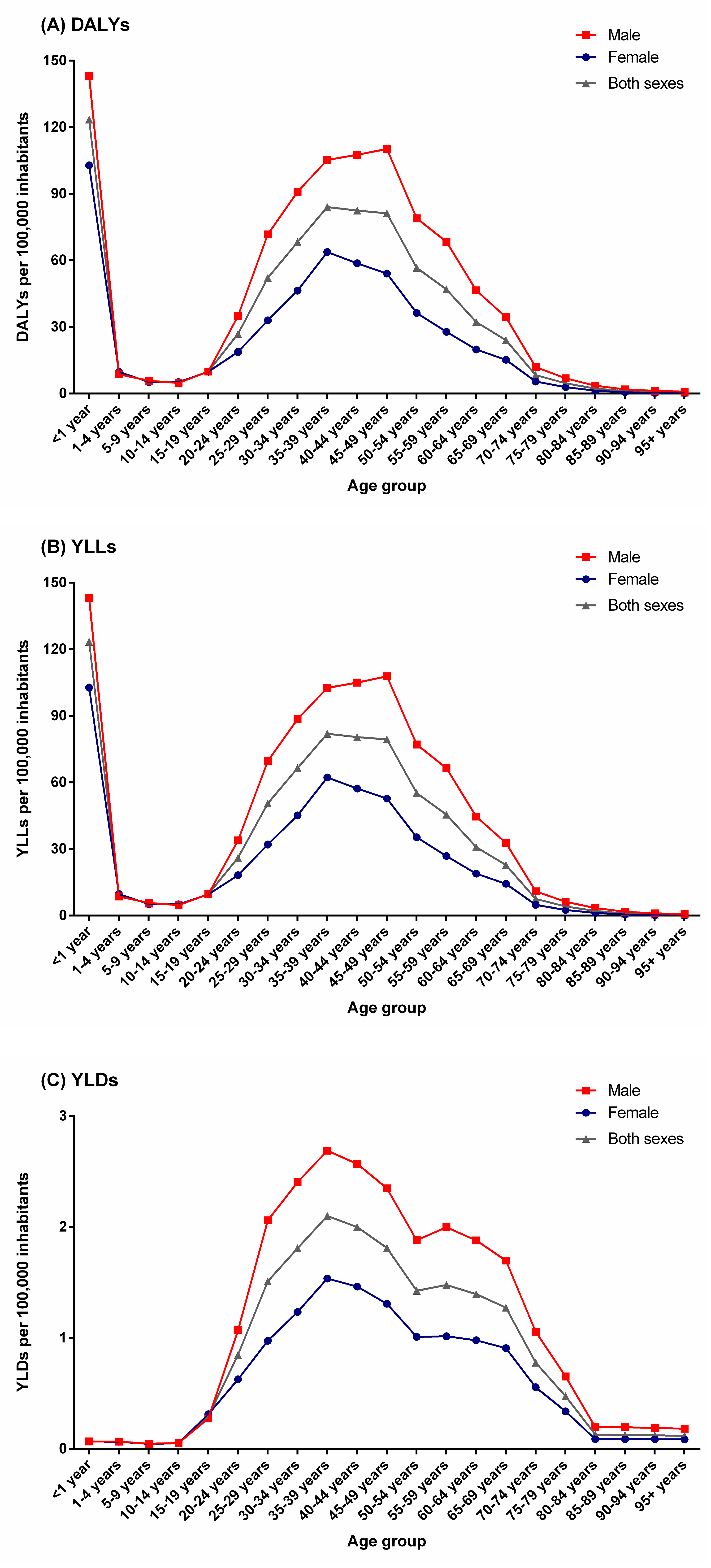

Supplement: Supplementary file 6 — Additional file 6: Figure S4. Age- and sex-specific rates (per 100,000 inhabitants) of (A) DALYs, (B) YLLs, and (C) YLDs from tuberculosis among HIV-positive individuals in Brazil, 2017. DALYs = disability-adjusted life-years; YLLs = years of life lost; YLDs = years lived with disability. [file 12963_2020_203_MOESM6_ESM.tif]

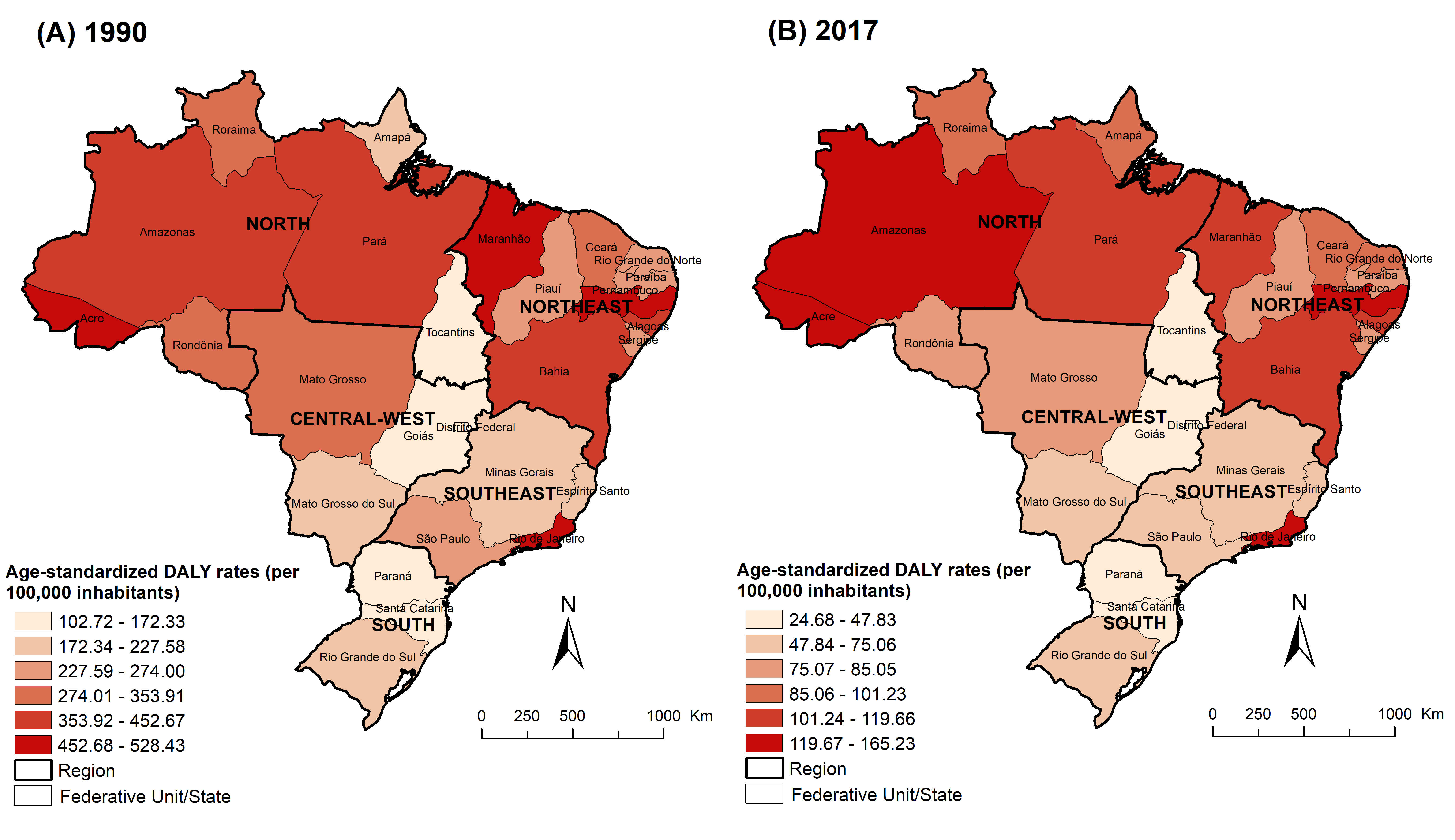

Supplement: Supplementary file 7 — Additional file 7: Figure S5. Age-standardized DALY rates (per 100,000 inhabitants) from tuberculosis among HIV-negative individuals by states in Brazil for 1990 and 2017. DALY = disability-adjusted life-years. [file 12963_2020_203_MOESM7_ESM.tif]

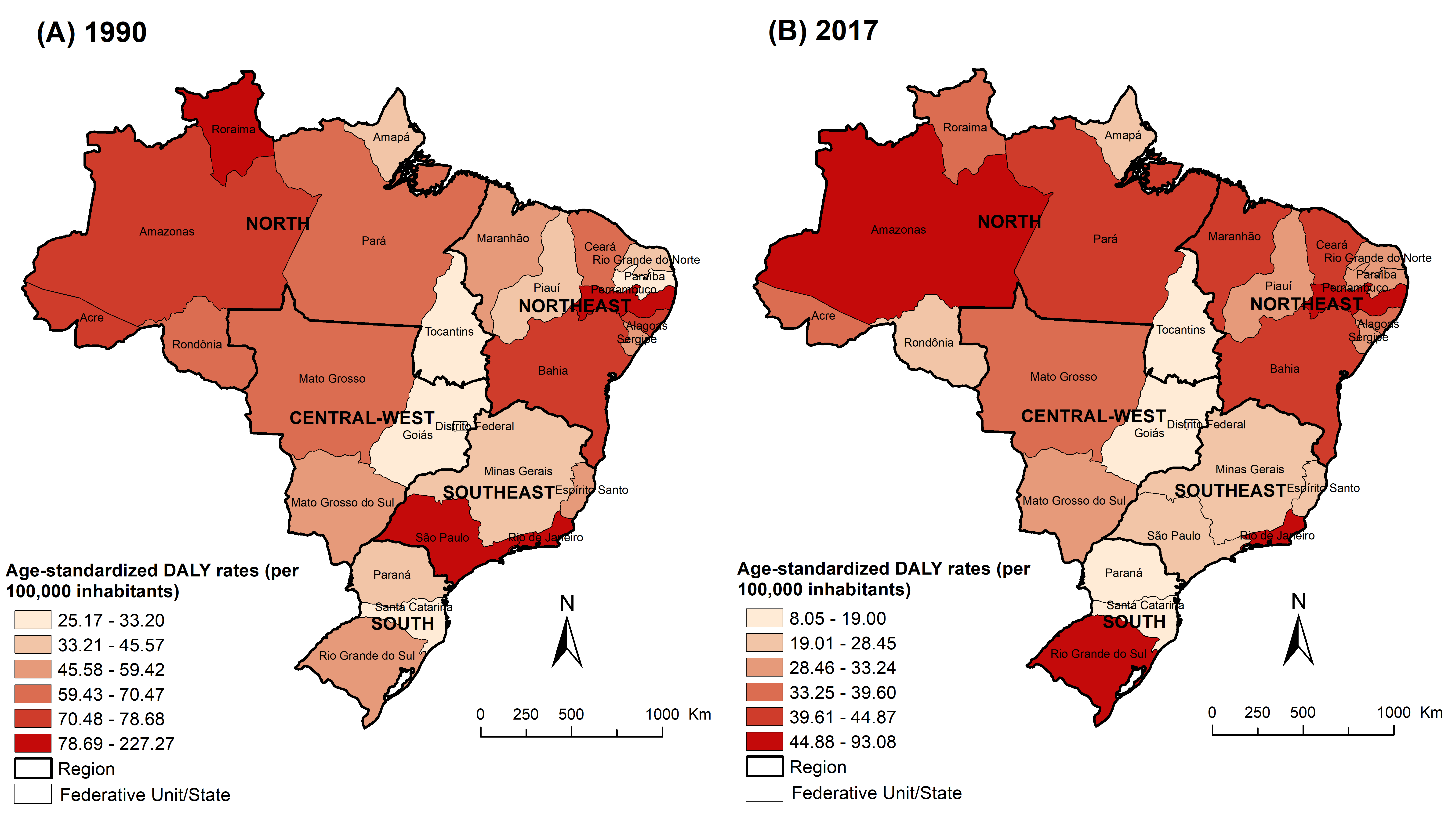

Supplement: Supplementary file 8 — Additional file 8: Figure S6. Age-standardized DALY rates (per 100,000 inhabitants) from tuberculosis among HIV-positive individuals by states in Brazil for 1990 and 2017. DALY = disability-adjusted life-years. [file 12963_2020_203_MOESM8_ESM.tif]
